# Unravelling Complex Hybrid and Polyploid Evolutionary Relationships Using Phylogenetic Placement of Homologous Gene Copies from Target Enrichment Data

**DOI:** 10.1093/sysbio/syag007

**Published:** 2026-01-27

**Authors:** Nora Walden, Christiane Kiefer, Marcus A Koch

**Affiliations:** Centre for Organismal Studies, Heidelberg University, Im Neuenheimer Feld 345, 69120 Heidelberg, Germany; Centre for Organismal Studies, Heidelberg University, Im Neuenheimer Feld 345, 69120 Heidelberg, Germany; Centre for Organismal Studies, Heidelberg University, Im Neuenheimer Feld 345, 69120 Heidelberg, Germany

**Keywords:** Angiosperms353, Arabideae, Brassicaceae764, phylogenetic placement, polyploid phylogenomics, target enrichment, whole-genome duplications

## Abstract

Phylogenomic data sets comprising hundreds of genes have become the standard for plant systematics and phylogenetics. However, large-scale phylogenomic studies often exclude polyploids and hybrids due to the challenges in assessing the origin of duplicated loci and incorporating them into tree reconstruction methods. Using a newly generated target enrichment data set of 1081 genes from 452 samples from the Brassicaceae tribe Arabideae, including many hybrid and high ploidy taxa, we developed a novel approach to disentangle the evolutionary history of this phylogenetically and taxonomically challenging clade. Our approach extends beyond commonly used gene tree-species tree reconciliation techniques by using phylogenetic placement, a method adopted from metagenomics, of gene copies into a diploid tree. We show how it allows for the simultaneous assessment of the origins of ancient and recent hybrids and autopolyploids, and the detection of nested polyploidization events. Additionally, we demonstrate how synonymous substitution rates provide further evidence for the mode of polyploidization, specifically to distinguish between allo- and autopolyploidization, and to identify hybridization events involving a ghost lineage. Our approach can serve as an exploratory tool for large and complex phylogenomic data sets and can aid in identifying polyploid and hybrid clades for further analysis with specialized methods.

Repeated cycles of polyploidization and diploidization in many plant lineages are considered a driving force behind the formation of new species (Soltis et al. 2009; Dodsworth et al. 2016), enhancing diversity and disparity (Clark and Donoghue 2018; Walden et al. 2020; Stull et al. 2021), and contributing to adaptation, radiation, and increased diversification rates (Soltis and Soltis 2016; Huang et al. 2020; but see Mayrose et al. 2011). The discovery of ancient whole-genome duplications (WGDs) in plants began with the detailed analysis of the *Arabidopsis thaliana* genome (Simillion et al. 2002; Bowers et al. 2003). Over the next 20 years, a multitude of lineage-specific WGDs across the green tree of life have been identified (Soltis and Soltis 2021; Qiao et al. 2022), typically discovered through sequencing entire genomes or transcriptomes (e.g., One Thousand Plant Transcriptomes Initiative 2019). Such WGDs may result from genome doubling within a single taxon (autopolyploidy) or hybridization between different taxa (allopolyploidy) (Burns et al. 2021), even across tribes (German and Friesen 2014; Mandáková et al. 2018; Walden et al. 2020). Distinguishing auto-from allopolyploidy is challenging, particularly for WGDs that occurred millions of years ago (Thomas et al. 2017; Zwaenepoel and Van de Peer 2019). Additionally, even for known and more recent hybrid origins, such as in allohexaploid *Brassica* (Chalhoub et al. 2014), phylogenetic trees often fail to accurately depict species’ evolutionary histories. This is because reticulate relationships, such as those in the evolution of allopolyploids, cannot be represented by a strictly bifurcating tree (Huson and Bryant 2006).

Phylogenetic reconstruction relies on the use of orthologs, gene sequences that diverged from each other due to speciation rather than gene duplication, which produces paralogs (Fitch 1970; Koonin 2005). Paralogs in this strict sense can be difficult to distinguish from duplicated genes that originated through speciation and were brought back together in a genome through allopolyploidy; these are referred to as homeologs (Glover et al. 2016). Orthology and homeology can be inferred through synteny (Walden and Schranz 2023) if whole-genome sequences are available, but are more challenging to detect in most phylogenomic data sets that lack complete genomes and detailed synteny information. Consequently, target enrichment studies frequently filter for single-copy loci either during the probe design phase (Johnson et al. 2019) or after sequencing (Jin et al. 2023; Scheunert et al. 2023). However, orthology is not guaranteed with single-copy loci. Firstly, differential gene loss (fractionation) following gene duplication or WGD can result in the inclusion of pseudoorthologs with single-copy genes (Xiong et al. 2022; Fedosov et al. 2024). Secondly, artifactual orthologs can arise when different copies of a multi-copy gene are assembled across samples, so that one sample contains one copy and another sample a different one, or when chimeric sequences are produced. Such mismatches lead to the recovery of hidden paralogs across taxa (Frost et al. 2024), particularly when data quality and sequencing depth are insufficient to assemble full length contigs of all copies. Thirdly, orthology inference based on sequence similarity may be biased by user-specified input parameters that cluster sequences into orthogroups. Additionally, single-copy gene sets often contain genes with conserved nucleotide sequences and gene functions that are under selection to revert to single-copy status after duplication (De Smet et al. 2013; Li et al. 2016; Walden and Schranz 2023). In allopolyploids, genes from one of the parental genomes are often preferentially retained, a pattern referred to as subgenome dominance (Cheng et al. 2018; Emery et al. 2018), introducing another bias that may dilute the signal of hybridization recoverable in phylogenomic data sets.

On the other hand, phylogenomic data sets, especially those employing data complexity reduction methods, like gene capture experiments, are powerful data sets to analyze very large sets of taxa across high phylogenetic distances (Zimmer and Wen 2015; Bartoš et al. 2023). A recent large angiosperm phylogenetic analysis (Zuntini et al. 2024) utilized the Angiosperms353 bait set (Johnson et al. 2019), capturing 353 nuclear single-copy genes and including 9506 species from 7923 genera across all angiosperm families. This study found that early angiosperm evolution at the order level is characterized by significant gene tree conflicts, in particular at nodes where WGDs are involved (Zuntini et al. 2024). Therefore, there remains a crucial need to unravel the phylogenetic information of homeologous genes and integrate them into phylogenetic trees. This integration is essential for reconstructing resolved species trees, or networks, that reflect the comprehensive evolutionary history of species, encompassing lineage divergence, genome duplication, and hybridization events (Oxelman et al. 2017; Rothfels 2021).

Inferring the evolutionary histories of polyploids from phylogenomic data sets is difficult because they contain multiple homeologous gene copies that must first be assembled correctly. Methods for obtaining such copies from target enrichment data phase read into alleles (Kates et al. 2018; Mendez-Reneau et al. 2023; Tiley et al. 2024), or use de novo assembly of divergent alleles (Johnson et al. 2016). In the case of allopolyploids, these multiple alleles further need to be phased into subgenomes. The different approaches available for this step (e.g., Nauheimer et al. 2021; Freyman et al. 2023) typically require some prior knowledge, such as potential parental species. Additionally, many of the studies using these methods are limited to small data sets with one or few well-defined hybridization events. This may in part also be due to the limitations posed on phylogenetic reconstruction caused by the reliance on bifurcating trees for large data sets, in which hybrid taxa cannot be properly displayed. Reticulate network analyses of phylogenomic data sets can account for hybridization between taxa (Jones et al. 2013; Solís-Lemus et al. 2017) and even extend the methodology to polyploids (Yan et al. 2022). However, computational limitations restrict such analyses to very small data sets (20–30 samples) and few hybridization events (Solís-Lemus and Ané 2016; Jin et al. 2023). Methods from population genetics to identify levels of introgression, such as D-statistics and fbranch analysis (Malinsky et al. 2021), have also been combined with species tree reconstruction and network analyses in smaller phylogenomic data sets such as in *Osmanthus* (Oleaceae; Li et al. 2023) and *Alchemilla* (Rosaceae; Morales-Briones et al. 2021). These approaches hold promise for improving the resolution and accuracy of phylogenetic trees and networks by integrating the complexities introduced by gene and WGDs. Nonetheless, these approaches still rely on a relatively resolved species tree and may be restricted to subsets of data.

Sequencing costs have decreased substantially in recent years, leading to the availability of larger data sets for plant systematics. However, appropriate analysis methods that can account for complex evolutionary patterns are still lacking. Highly polyploid taxa, which may have undergone multiple, nested WGDs, are rarely analyzed in their full complexity. Instead, their analysis is often simplified by using what appear to be strict single-copy loci, or by summarizing copies into consensus sequences in phylogenetic studies (Hendriks et al. 2023). An additional confounding factor is that the ploidy status of many plant species remains unknown and cannot easily be determined using available herbarium vouchers, which may be the only material accessible for study.

To contribute to ongoing efforts toward addressing these challenges, we developed an exploratory approach that incorporates gene copies assembled from target enrichment data into the reconstruction of an evolutionary hypothesis using “phylogenetic placement,” a method commonly used in metagenomics (Czech et al. 2022). This method requires no prior knowledge of hybridization, WGD, or ploidy status. Importantly, it also does not require that paralogs (copies that originated though gene duplication) and homeologs (copies originated through genome duplication) be differentiated from orthologs. We apply this approach to a data set of 452 samples from the Brassicaceae tribe Arabideae. Arabideae is the largest tribe within Brassicaceae, containing 559 of the family’s 4000 species in 18 genera (German et al. 2023), including the largest genus (*Draba*) with ~400 species (Karl and Koch 2013). The tribe is distributed primarily in Eurasia and North America, with fewer species in Africa and South America.

*Arabis alpina*, the taxonomic type of the tribe, has served as a model system for studying annual/perennial lifestyle (Kiefer et al. 2017; Wötzel et al. 2022) and flowering time (Madrid et al. 2021). Several genomes from the tribe have been sequenced in recent years, providing valuable models in comparative genomics (Willing et al. 2015; Nowak et al. 2021). The Arabideae contain a high number of (neo)polyploid species, about 63%, compared to approximately 43% across the entire Brassicaceae (Hohmann et al. 2015), making it taxonomically challenging (Karl and Koch 2013). However, most major clades within Arabideae have been extensively studied, providing solid background information on taxonomy, biogeography, and evolutionary history (Koch et al. 2006, 2010, 2012, 2017; Jordon-Thaden et al. 2010; Karl et al. 2012; Karl and Koch 2013, 2014). This research highlights substantial hybridization, reticulation, and introgression on various spatiotemporal scales, from closely related species and timescales of a few hundred thousand years (Koch et al. 2010; Koch and Grosser 2017) to intercontinental merging of gene pools giving rise to new evolutionary lineages millions of years ago in the giant genus *Draba* (Jordon-Thaden et al. 2010). Given the high prevalence of polyploidy and the potentially complex history of lineage divergence and hybridization, this Arabideae data set is an ideal candidate to test our new approach to unravel evolutionary histories using target enrichment data.

## Materials and Methods

### Taxon Sampling

Plant material from across tribe Arabideae (supertribe Arabodae) and outgroup taxa from tribes Boechereae and Turritideae (supertribe Camelinodae) and Fourraeeae (supertribe Brassicodae) was collected from herbarium specimens, silica-dried leaf material, and DNA stocks from samples included in previous studies (Supplementary Table S1). DNA stocks were available from 149 samples from previous studies (Koch and Al-Shehbaz 2002; Jordon-Thaden et al. 2010; Koch et al. 2010, 2017; Karl et al. 2012; Karl and Koch 2013; Kiefer et al. 2017). In total, we included 452 samples, eight of them with sequencing replicates, representing 382 accepted species, two of which were additionally represented by two subspecies (63 *Arabis* species, 264 *Draba* species, 19 *Aubrieta* species, and 36 other species), which included also five samples from our previous Brassicaceae-wide study (Hendriks et al. 2023). Further information on specimens and vouchers is available in Supplementary Table S1.

Chromosome counts for 179 Arabideae taxa from our data set were extracted from *BrassiBase* (Kiefer et al. 2014, 2025; Koch et al. 2018), and count data for an additional 19 taxa were added from PloiDB (Halabi et al. 2023). Base chromosome number in the tribe is eight (Hohmann et al. 2015), though many published chromosome counts are not multiples of eight, suggesting descending dysploidy to five to seven chromosomes (Mandáková et al. 2020). We classified chromosome number into three classes (“eight,” “not eight,” and “both eight and not eight” for taxa with different reported chromosome numbers) and ploidy into eight classes (“diploid,” “tetraploid,” “hexaploid,” “octoploid,” “dodecaploid,” “hexadecaploid,” “low,” and “high”; where low and high were assigned to taxa with multiple reported ploidy levels with average ploidy below and above tetraploid, respectively).

Formal new taxonomic recognition and description of one taxon, *Turritis subflava*, was necessary and is presented with Supplementary Text.

### DNA Isolation, Target Enrichment, Library Preparation, and Sequencing

The samples obtained from herbarium specimens were ground using a Precellys24 homogenizer (PEQLAB Biotechnology GmbH, Erlangen, Germany). DNA was then extracted using the Invisorb Spin Plant Mini Kit (Invitek-Molecular GmbH, Berlin, Germany) according to the manufacturer’s protocol. DNA was eluted in 50 µL elution buffer. DNA concentration was measured by Qubit (dsDNA HS Assay Kit, Invitrogen/Thermo Fisher Scientific, Waltham, MA, USA) and DNA integrity was assessed by electrophoresis (1% TAE agarose gel).

Prior to library preparation, the integrity of DNA was checked again on an Agilent Tape Station (Agilent Technologies GmbH, Darmstadt, Germany) using the genomic DNA screen tape and corresponding genomic DNA reagents. Fragmentation was only applied if DNA fragments were >500 bp. For fragmentation, the samples were diluted with water to obtain 200 ng of DNA in a volume of 60 µL. Fragmentation was performed using Covaris micro Tubes AFA Fibre with pre-slit SnapCap (6 × 16 mm; Covaris Ltd, Brighton, UK) in a Covaris ultrasonicator (Covaris Ltd, Brighton, UK) for 80 s (high fragmentation) or 60 s (low fragmentation).

Libraries were prepared using the NEBNext Ultra II DNA Library Kit (New England Biolabs GmbH, Frankfurt, Germany) with purification beads, and 100 ng of the fragmented/non-fragmented DNA in a volume of 25 µL was used as input according to the manufacturer’s protocol with some alterations. For the NEBNext End Prep (New England Biolabs GmbH, Frankfurt, Germany), half the amount of chemicals was used. For adapter ligation, the adapters were diluted 1:15 and half of the amount stated in the protocol was used. Fragments were size-selected based on the beads included in the kit (400–500 bp).

For the PCR enrichment of adapter-ligated DNA, the forward and reverse primer were already combined and NEBNext Multiplex Oligos (New England Biolabs GmbH, Frankfurt, Germany) for Illumina Set 1 to 3 were used as dual index primer pairs in a PCR with 15 cycles according to the manufacturer’s protocol. The PCR reactions were cleaned up using NEBNext sample purification beads (New England Biolabs GmbH, Frankfurt, Germany). Concentration of the libraries was determined by Qubit using the Qubit dsDNA HS Assay Kit (Invitrogen/Thermo Fisher Scientific, Waltham, MA, USA) and library size was measured on an Agilent Tape Station using D1000 Screen Tape (Agilent Technologies GmbH, Darmstadt, Germany).

In a next step, libraries were pooled (32–43 libraries; a total of 12 pools). Pool 1 was size selected by E-Gel (Thermo Fisher Scientific, Waltham, MA, USA). Pools were then purified using Ampure XP beads (Beckman Coulter Life Sciences, Krefeld, Germany), the concentration was measured again by Qubit dsDNA HS Assay Kit (Invitrogen/Thermo Fisher Scientific, Waltham, MA, USA), and size of the pools was determined by Agilent Tape Station using Agilent Tapestation D1000 Screen Tape (Agilent Technologies GmbH, Darmstadt, Germany).

For hybridization capture, two bait sets (Angiosperms353 and a custom bait set for Brassicaceae, hereafter called Brassicaceae764, Arbor Sciences) were used as described previously (Hendriks et al. 2021). Baits were mixed 2:1 (Angiosperms353:Brassicaceae764) and hybridization was carried out at 60 °C for 24 h in a PCR cycler. Binding to streptavidin-coated magnetic beads and washing of the reactions for removal of non-target DNA were performed according to the manufacturer’s protocol (Arbor Scientific, Arbor Biosciences, Ann Arbor, MI, USA). In a last step, libraries were suspended and amplified using NEBUltra Q5 polymerase (New England Biolabs GmbH, Frankfurt, Germany), IDT xGen (IDT, Coralville, IA, USA) amplification primer; 18 cycles; all according to manufacturer’s protocol. Enriched pools were purified using Ampure XP beads (Beckman Coulter Life Sciences, Krefeld, Germany). Reamplification of the purified PCR product was performed for 10 cycles using the same polymerase and primer as stated above. Final purification was done using Ampure XP beads (Beckman Coulter, Krefeld, Germany). Concentration of the pools was determined by Qubit (DNA HS Assay, Invitrogen/Thermo Fisher Scientific, Waltham, MA, USA) and pools were checked on an Agilent Tape Station (D1000 Screen Tapes, D1000 reagents, Agilent Technologies GmbH, Darmstadt, Germany). Sequencing was performed at Novogene UK by Illumina sequencing technology (150 bp paired end reads; 48–65 GB data per pool) using 25 µL of each pool. All raw reads are available at ENA (https://www.ebi.ac.uk/ena/browser/home) under accession PRJEB72669.

### Processing of Target Enrichment Data

Raw sequencing reads were processed with Trimmomatic 0.39 (Bolger et al. 2014) to remove remaining adapter sequences and trim reads for quality with leading and trailing quality 20, a quality of 20 in a sliding window of 4 bp, and minimum length 30. Next, we tested whether sequence-based ploidy inference could be performed on target enrichment data. As we expected our data set to contain taxa with high ploidy based on literature, Smudgeplot (Ranallo-Benavidez et al. 2020) was the only suitable option. Sequence-based ploidy inference was conducted on the trimmed reads. KMC 3.1.1 and kmc_tools 2.3.0 (Kokot et al. 2017) were used to calculate kmer frequencies and generate a histogram of kmers using kmer length 17 and a maximum count of 10,000. Smudgeplot was then used to infer ploidy level using a lower coverage limit of 4 and an upper limit estimated from the kmer histogram. Note that although this software can infer ploidy levels from raw reads of highly polyploid samples, it was designed for whole-genome rather than target enrichment data.

Our data analysis pipeline for the following steps is illustrated as a flowchart in Figure 1. Assembly of gene sequences was conducted with HybPiper 2.0 (Johnson et al. 2016) using the 1081 target genes as a reference (Johnson et al. 2019; Nikolov et al. 2019). As the two bait sets were developed independently, some genes were contained in both. Following Hendriks et al. (2023), these 36 genes were removed from the Brassicaceae764 set, as the sequences in this set were generally shorter than those of the Angiosperms353 set. We ran the HybPiper command “hybpiper assemble” in nucleotide mode using bwa for read mapping and subsequently obtained assembly statistics with “hybpiper stats.” Due to the high mapping rate (Supplementary Fig. S1), we could apply a strict quality threshold and only select samples with at least 900 genes covered (GenesWithContigs ≥ 900) and at least 50% of genes covered at 50% length (GenesAt50pct ≥ 226) in HybPiper (Supplementary Fig. S2) in a first filtering step. In a second step, only genes with less than 20% of missing samples were retained for further analysis (Supplementary Fig. S3). The final data set comprised 442 samples and 994 genes. We then obtained all gene copies for the selected samples using “hybpiper paralog_retriever” in “gene” mode (i.e., without introns). Potential chimeric contigs identified by HybPiper were excluded from further analysis. Because the HybPiper identification of chimeric contigs may miss some chimeric assemblies, we also tested our downstream analyses on a set of 567 exons with a minimum length of 500 bp as well as the highest number of variable sites and bipartition support among exons of each gene. Stitched contigs containing multiple de novo assembled contigs were excluded from this analysis.

**Figure 1.**
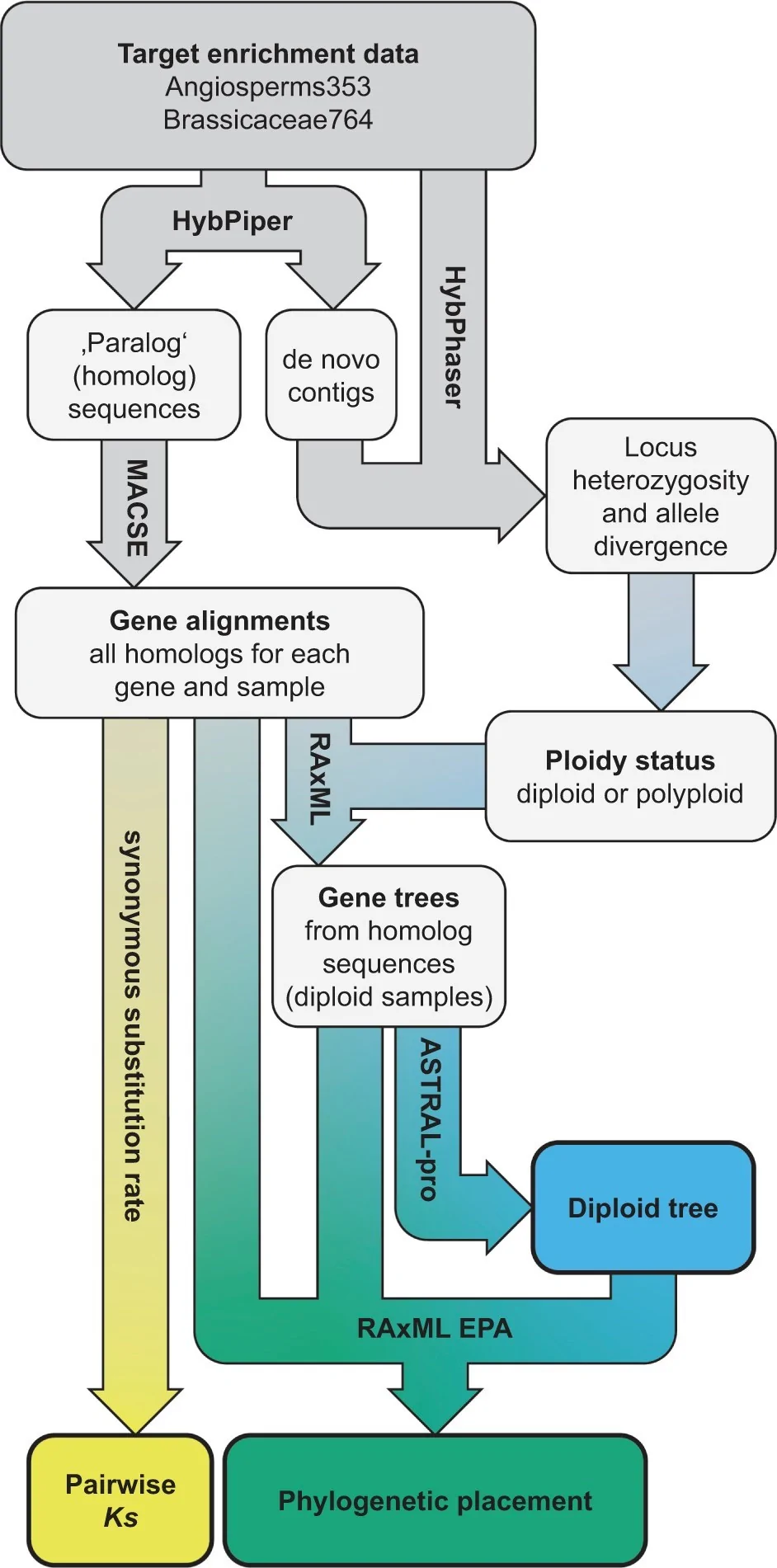
Flowchart of our data analysis pipeline. Target enrichment data was processed with HybPiper to obtain homolog sequences, which were aligned and subjected to gene tree reconstruction. Using locus heterozygosity and allele divergence from HybPhaser, ploidy status was inferred to obtain subsets of homolog sequences containing only “diploid” samples; the diploid tree was reconstructed using these alignments. Pairwise Ks values were obtained from the same alignments. Finally, evolutionary placement of homolog sequences into the diploid tree was performed using RAxML-EPA.

Next, we assessed whether multiple copies of each target gene were present in the assembled sequences. The first steps of the HybPhaser 2.0 (Nauheimer et al. 2021) pipeline (scripts 1_generate_consensus_sequences.sh and 1b_assess_dataset.R) were run to generate consensus sequences and obtain allele divergence (the percentage of single nucleotide polymorphisms (SNPs) across all loci) and locus heterozygosity (the percentage of loci that contain SNPs) of all remaining samples and genes using a minimum depth for inserting ambiguity codes of 8, minimum allele frequency of 0.1, and minimum count for alleles to be considered for assigning ambiguity code of 3.

### Phylogenetic Reconstruction

As we expected high levels of polyploidy, we used a multi-step approach to reconstruct the evolutionary history of tribe Arabideae. We split the data set into “diploid” and “polyploid” individuals based on allele divergence <2% and locus heterozygosity <90% obtained from HybPhaser (hereafter referred to as “ploidy status”). Ploidy level information from the literature was available for 198 taxa (corresponding to ~52% of the accessions in our study); these ploidy levels were largely in line with our HybPhaser-based ploidy status classifications (Fig. 2a). It is important to note that this classification does not necessarily reflect the true ploidy level according to chromosome number—a “diploid” ploidy status may be inferred for autopolyploids due to low heterozygosity, and conversely, a “polyploid” status may be inferred for homoploid hybrids or for old allopolyploids that subsequently underwent rediploidization, due to high heterozygosity. Ploidy status inferred using this method was highly inconsistent with kmer-based ploidy level inference using smudgeplot (Fig. 2c). Although this software can infer ploidy levels from raw reads of highly polyploid samples, it assumes genome-wide, uniform coverage, and therefore is not appropriate for target enrichment data. Because literature data were only available for 246 of 442 samples, we used the HybPhaser-based ploidy status in the following analyses and classified 141 samples as “diploid.”

**Figure 2.**
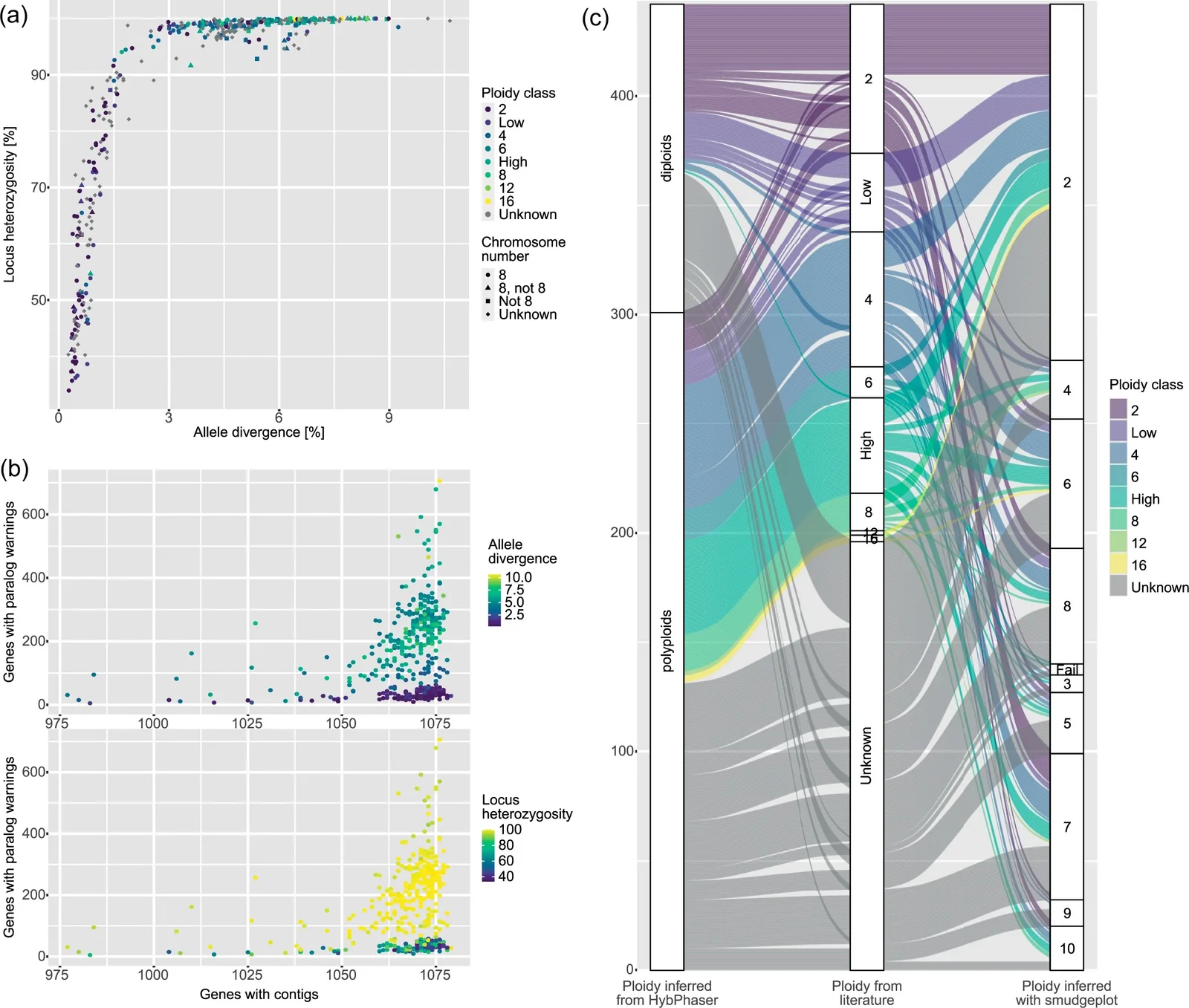
Ploidy assessment. (a) Allele divergence (AD) and locus heterozygosity (LH) from HybPhaser are shown for all samples together with ploidy level and chromosome number from the literature. Samples with lower ploidy level (shown in darker colors) tend to have lower AD and LH than samples with high ploidy level (lighter colors), in line with expectations; (b) number of genes with paralog warnings by length from HybPiper and number of genes with contigs. AD (upper panel) and LH (lower panel) of the different homologs within each sample as obtained by HybPhaser are plotted against the number of paralog warnings from HybPiper and the number of contigs with sequences. Samples that failed our minimum sequence recovery threshold of 900 genes and genes that failed our minimum sequence recovery threshold of less than 20% missing samples were excluded from the analysis; (c) comparison of the three ploidy inference methods used here. Ploidy level from the literature is shown in the center, inference from HybPhaser data using AD <2 and LH <90 as a threshold to define diploids, resulting in two groups (“diploids” and “polyploids”), is shown on the left, and ploidy inference from sequencing data using kmers in smudgeplot is shown on the right. Samples assigned to the “diploids” group using HybPhaser contained mostly diploid taxa or those with low ploidy levels according to literature, whereas most tetraploid and hexaploid taxa and all samples with known high ploidy (>hexaploid) were classified as “polyploids.” Conversely, ploidy assignment using smudgeplot was largely inconsistent with ploidy reported in literature, though interestingly many known diploids assigned to “polyploids” using HybPhaser were also inferred to be polyploid.

Separate nucleotide sequences of all gene copies were obtained by HybPiper for each gene (366–1146 sequences per gene, mean number of sequences per gene 520; 916–1719 sequences per sample, mean number of sequences per sample 1170). We used only sequences that passed the chimeric sequence detection in HybPiper and aligned them with mafft 7.511 (Katoh and Standley 2013) using the FFT-NS-2 method, followed by realignment with refineAlignment in MACSE 2.06 (Ranwez et al. 2018) to obtain codon-aware nucleotide alignments. Alignments were trimmed with trimAl 1.4.rev22 (Capella-Gutiérrez et al. 2009).

Subsequently, sequences from diploid individuals were extracted from the initial alignment for separate analysis. The subset gene alignments were trimmed with trimAl, and maximum likelihood (ML) gene trees were inferred with RAxML v8.2.12 (Stamatakis 2014) using the GTRCAT substitution model, with 1000 rapid bootstrap inferences followed by a thorough ML search. As the diploid data set contained multiple copies for some genes (number of copies per locus per sample: min 0.9215, median 1.0312, mean 1.0285, max 1.0644), we used ASTRAL-pro 1.13.1.3 (Zhang et al. 2020) to combine the gene trees into a species tree (subsequently called “diploid tree”).

To infer the phylogenetic position of each gene copy from polyploid samples, diploid gene trees were first rooted to the two diploid outgroup species from tribe Boechereae using nw_reroot from Newick utilities (Junier and Zdobnov 2010); in cases where one or both of the outgroup samples were missing or had multiple sequences, trees were rooted manually in figtree 1.4.4 (http://tree.bio.ed.ac.uk/software/figtree/). Genes with a high proportion (≥10%) of missing samples among diploids were discarded, as were genes with a high proportion (≥10%) of samples with multiple copies, resulting in 857 genes (Supplementary Fig. S4). Genes with high proportions of multiple copies often contained two mirrored subtrees, and were likely retained from the Brassicaceae-specific At-α WGD (Barker et al. 2009). Phylogenetic placement of gene copies into the diploid gene trees was then conducted using RAxMLs evolutionary placement algorithm (EPA; Berger et al. 2011) with the same substitution model as above for each gene (Fig. 1). The EPA is particularly useful for our approach, because query sequences, in our case the different gene copies, can be placed at any edge within the reference tree, not only the terminal edges, as the algorithm does not rely on pairwise similarity with extant sequences. Our data set contains polyploids for which the history of WGDs is unknown and could be complex, with multiple and even nested hybridization events between lineages giving rise to entire clades. Using phylogenetic placement of gene copies therefore allowed us to pinpoint the placement of the last common ancestor of the extant gene copy and the diploid species, and ancient autopolyploidization events as well as hybridization could be inferred from the emerging patterns. RAxML’s EPA outputs the likelihood of placements into each branch as likelihood-weight ratios (lwrs), and we added up the lwr of all placements of all copies across each sample and subsequently normalized branch-specific lwrs by the total sum of lwrs per sample across all 857 genes to account for uneven numbers of sequences recovered across taxa. This resulted in a unique placement pattern for each “polyploid” sample, though many samples showed similar patterns. We used hierarchical clustering of the normalized placement to group samples with similar patterns, which seem to share sequence placement potentially indicative of common ancestry. For each cluster, we then considered only placements into branches reaching a mean lwr of at least 0.05 across all samples for visualization in the diploid anchor tree. This removes signal from single-gene duplications as well as low gene tree resolution, while leaving in only the branches that seem to have played a major role in the evolutionary history of the particular cluster. Filtering of data sets was performed in R 4.2.2 (R Core Team 2022) using stringr 1.5.0 (Wickham 2022). Phylogenetic trees were read with treeio 1.22.0 (Wang et al. 2020) and ape 5.7-1 (Paradis and Schliep 2019), and visualized using ggtree 3.6.2 (Yu et al. 2017). Additional tree information was extracted with phytools 1.9-16 (Revell 2012) and MoreTreeTools 0.0.1 (Bennett 2014). Phylogenetic placement results were read using BoSSA 3.7 (Lefeuvre 2020). Clusters were extracted from dendrograms with ggdendro 0.1.23 (Vries and Ripley 2022). Figures were generated with ggplot2 3.4.2 (Wickham 2016); ggpointdensity 0.1.0 (Kremer 2019) for point density scatterplots; viridis 0.6.3 (Garnier et al. 2023) for color scales; ggExtra 0.10.1 (Attali and Baker 2023) for marginal histograms; ggalluvial 0.12.5 (Brunson and Read 2019) for the alluvial diagram; and ggpubr 0.6.0 (Kassambara 2023) for arranging multi-panel figures. All data are available at Dryad (https://doi.org/10.5061/dryad.xksn02vqn).

### Analysis of Synonymous Substitution Rate of Gene Copies

We further analyzed sequence placement patterns of samples from two clusters as examples for different evolutionary scenarios (cluster 1: hybridization between diploid lineages in *Draba*; cluster 30: polyploid origin of *Arabis nordmanniana* and relatives) using pairwise synonymous substitution rates (*Ks*) between all copies within polyploid samples and between polyploids and their diploid relatives. Alignments were read and *Ka*/*Ks* values calculated with seqinr 4.2-36 (Charif and Lobry 2007). Taxa of interest, as well as three outgroup taxa to assess consistency and reliability of the method, were analyzed for each cluster and results were displayed with ggridges 0.5.4 (Wilke 2022).

### The Genus *Arabidopsis* as a Test Data Set

To test our approach on polyploid samples of known origin, we used whole-genome resequencing data from the genus *Arabidopsis*. The genus is particularly useful for this purpose, as its evolutionary history is well studied using genomic data and it contains diploids and both auto- and allopolyploids (e.g., Hohmann et al. 2014; Novikova et al. 2016; Koch 2018; Kiefer et al. 2023). The *A. arenosa* clade consists of multiple diploid and autopolyploid taxa (Schmickl et al. 2012; Arnold et al. 2015), *A. suecica* is an allotetraploid that originated from hybridization of *A. arenosa* and *A. thaliana* (Sall et al. 2003; Jakobsson et al. 2006; Novikova et al. 2017; Burns et al. 2021), *A. kamchatica* is an allotetraploid that originated from hybridization of *A. halleri* and *A. lyrata* (Shimizu-Inatsugi et al. 2009), and finally tetraploid *A. lyrata* shows introgression from *A. arenosa* (Schmickl and Koch 2011; Hohmann and Koch 2017).

Whole-genome resequencing data from a genus-wide study was downloaded from ENA for a subset of 24 samples covering all evolutionary lineages, including eight tetraploids (see Supplementary Table S2 for details). The raw reads were trimmed and processed following our pipeline outlined above, and ploidy detection using HybPhaser, phylogenetic reconstruction, and phylogenetic placement of gene copies were performed using the same 994 genes as selected for the Arabideae data set.

## Results

### Data Quality Is Very High for Selected Genes and Samples

We obtained high read mapping rates for most newly sequenced samples (2.4–82.3%, mean 66.52%, median 70.7%; see Supplementary Fig. S1) despite including a number of old herbarium vouchers dating back as far as 1823. This also led to generally high rates of gene recovery (977–1079 genes with contigs, mean 1067, median 1071; 809–1074 genes at 50%, mean 1002, median 1001); 10 samples with fewer than 900 genes with contigs and fewer than half of all genes covered at 50% were excluded from further analysis (Supplementary Fig. S2). Similarly, the high gene recovery rate allowed us to apply a fairly strict threshold for number of missing samples in each gene (20%) while only excluding 87 genes from downstream analyses (Supplementary Fig. S3). The final data set thus consisted of 994 genes from 442 samples.

### Multiple Copies Are Common and Many Taxa Are Polyploid

Many selected samples had high numbers of paralog warnings in HybPiper (5–706 paralogs, mean 159.1, median 140). The highest number of copies, both with regard to number of genes with multiple copies (659) and total sequences (1718), was recovered for polyploid *Draba ladyginii*, whereas *Draba linearifolia* had the lowest number of genes with multiple copies (5).

Detection of multiple gene copies can be influenced by coverage, specifically by the number of reads mapped to the target, as the multiple gene copies are assembled de novo. In our data set, multiple copies were recovered in known polyploid samples even with relatively low numbers (>200,000) of mapped reads (Supplementary Fig. S5a). In line with expectations, we detected lower copy numbers in diploid taxa (ploidy inferred from literature) and those with low ploidy (i.e., taxa with multiple but low ploidy levels reported in the literature) compared to polyploids (Supplementary Fig. S5b). Additionally, allele divergence (AD) and locus heterozygosity (LH) were generally high in known polyploid taxa (Fig. 2). Samples assigned to the “diploids” group using HybPhaser (AD < 2 and LH < 90; see Fig. 2) contained mostly diploid taxa or those with low ploidy levels according to literature, whereas most tetraploid and hexaploid taxa and all samples from taxa with reported high ploidy (>hexaploid) were classified as “polyploids” (Supplementary Fig. S5). Conversely, ploidy assignment based on raw reads using smudgeplot was largely inconsistent with ploidy reported in literature (Fig. 2c). Interestingly however, most samples from diploid taxa according to the literature, which were assigned to “polyploids” using HybPhaser, were also inferred to be polyploid using smudgeplot, likely due to their high heterozygosity. These samples could represent previously unreported polyploid cytotypes from diploid taxa, homoploid hybrids with high heterozygosity, or rediploidized ancient allopolyploids that have reverted to a diploid chromosome number but retained multiple gene copies. With regard to the number of copies detected, we found a strong positive correlation with assembly quality (i.e., number of sequences assembled in a single contig), suggesting that copies may have been missed in “polyploid” samples with lower assembly quality, or assembled into chimeric contigs (Supplementary Fig. S6).

### The Diploid Tree Is Reliable and Suitable for Phylogenetic Placement

We inferred a phylogenetic backbone of Arabideae from 141 “diploid” samples identified using HybPhaser (Fig. 2). Note that as sequence divergence between alleles was the prerequisite for ploidy determination, polyploids showing low copy divergence, such as autopolyploids, may be classified as diploids using this method; conversely, homoploid hybrids, despite having diploid chromosome numbers, are likely classified as polyploids. The diploid tree reconstructed using ASTRAL-pro based on the 994 genes recovered the major clades of Arabideae: the genus *Draba, Arabis aucheri*, genus *Aubrieta*, the *Arabis alpina* clade, main *Arabis* clade, and *Scapiarabis* clade (Supplementary Fig. S7). The *Tomostima* clade as well as *Erophila*, and *Drabella* consisted exclusively of “polyploids” and were therefore not included. Support values in the tree, in particular at backbone nodes, were high, with local posterior probabilities largely >0.95 and relatively high quartet scores (Supplementary Fig. S8), indicating low conflict and high resolution at these nodes in the diploid tree.

### Phylogenetic Placement into the Diploid Tree Identifies Patterns and Evolutionary Processes

The diploid gene trees and species tree were used as the basis for the next step, phylogenetic placement of gene copies (blue in Fig. 1). The pattern of placement likelihood-weight ratios (lwrs) showed clade-specific clustering in particular branches of the tree (Figs. 3; Fig. S9). Note that short internal branches lead to low lwrs at multiple nearby nodes, thus pinpointing the exact branch for each placement was not always possible. Hierarchical clustering of the placement patterns into 43 clusters (Figs. S10 and S11) was used to summarize taxon groups with potentially similar evolutionary histories (Figs. S12-–S22), either through independent or shared origin.

**Figure 3.**
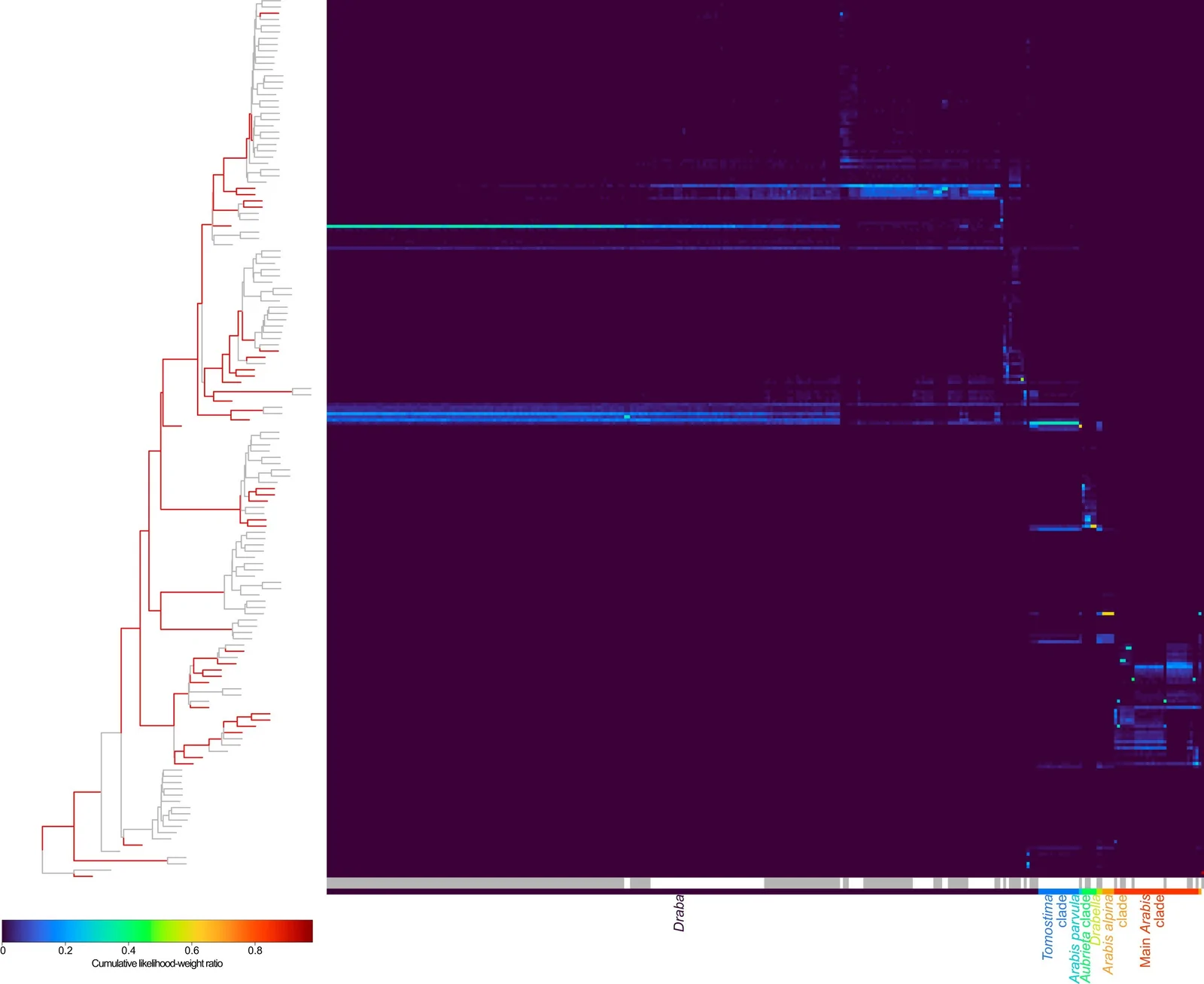
Placement of homologs. Branches with a cumulative likelihood-weight ratio (lwr) of greater than 0.05 in any cluster are highlighted in the diploid tree on the left side; some clades are involved in notably few polyploidization/hybridization events. The heatmap on the right shows lwrs for all polyploid samples (*x*-axis) across the branches of the diploid tree (*y*-axis). Similar patterns of lwr across species indicate their similar (parallel) or shared evolutionary history; the 43 clusters of similar patterns are highlighted below the heatmap. For the same figure with full taxon labels, see Supplementary Fig. S9.

Bringing together the placement patterns and the anchoring diploid tree for each sample, we could identify different evolutionary processes based on the number of distinct branches with placement: A majority of gene copies within a sample placed into a single branch indicates a scenario where all gene copies within a polyploid share their most recent common ancestor along this branch, such as an autopolyploidization event. This pattern was observed in samples summarized as cluster 25 (Supplementary Fig. S18), cluster 28 (Supplementary Fig. S18), and cluster 30 (Supplementary Fig. S19).

Gene copies within polyploids mapping to two distinct branches indicate hybridization or allopolyploidization (e.g., samples in cluster 1; Supplementary Fig. S12). Different copy numbers mapping to both involved branches could be a result of asymmetric gene loss after hybridization, of introgression, or polyploidizations involving uneven numbers of chromosome sets from the parental species. Placement patterns consistent with hybridization were found in many samples in our study, among them those summarized in clusters 1–3 (Supplementary Fig. S12) and clusters 31–37 (Figs. S19–S21). The presence of additional WGDs or hybridization events can lead to placement patterns involving more than two distinct branches in the diploid tree, such as in samples from cluster 4 (Supplementary Fig. S12). These samples show similar placement patterns as those from clusters 1–3, but with additional copies mapping to a third branch, indicating subsequent exchange of genetic material with a third lineage.

Finally, we observed some samples in which gene copies were placed to many different, neighboring branches along the phylogenetic backbone, for example *Drabella muralis* (Supplementary Fig. S19). This pattern may be due to homoplasy or a rapid divergence of the major lineages, leading to a lack of nucleotide differences at the targeted genes at the backbone nodes of the Arabideae phylogeny and thus low gene tree resolution. To a lesser extent, this pattern was also observed in other samples, such as in the species in cluster 30 (Supplementary Fig. S19), where it could indicate that the inferred WGD occurred shortly after lineage divergence, whereas in other cases it may simply be indicative of the rapid diversification in the tribe leading to incomplete lineage sorting.

The analysis of individual exons instead of longer gene sequences resulted in largely congruent results. The diploid tree (Supplementary Fig. S23) showed the same topology as the one based on the full target sequences with similarly high support. Finally, the placement of the shorter exon copies resulted in the same placement patterns as using gene sequences (Supplementary Fig. S24).

### Synonymous Substitution Rates Indicate Evolutionary History and Relative Age Estimates

Synonymous substitution rates between homeologs can be used to elaborate on the inferred/hypothesized evolutionary scenarios, as well as to provide some relative estimates for the time of polyploidization and hybridization. Here, we chose two exemplary clusters as study cases and analyzed them in more detail using pairwise *Ks* within polyploid samples and between polyploid and diploid relatives. Samples from clusters representing two different evolutionary scenarios for the origin of hybrids and polyploid taxa (see Supplementary Fig. S25), namely the formation of homoploid or polyploid (allopolyploid) hybrids, as well as potential autopolyploidization, were chosen.

The first example highlights hybridization among two diploid lineages, which hybridized after their divergence and gave rise to a polyploid taxon, which afterward may undergo further lineage diversification. This is the case in samples of cluster 1 (Fig. 4), where gene copies were placed at the branches of two diploid clades, *Draba polytricha* and the clade of *D. lutescens, D. huetii*, and *D. ellipsoidea*. We analyzed pairwise within- and between-sample *Ks* for selected diploids (*D. polytricha* and *D. lutescens*) and polyploids (*D. exunguicula* and *D. mogollonica*), as well as outgroups (Fig. 4). Gene copies from the polyploid samples mapping to the two different branches of the diploid tree (Fig. 4b) were analyzed separately here. Within-polyploid *Ks* (i.e., *Ks* between copies) were similar in both polyploid representatives. Furthermore, within-polyploid *Ks* were higher than those observed in pairwise comparisons with the closer diploid (Fig. 4c; closer diploid refers to the taxon that the respective homeolog mapped to during the placement analysis) and higher than pairwise *Ks* with the close homeolog from the other polyploid, in line with a scenario where two diploid lineages hybridized sometime after their divergence and their hybrid offspring subsequently underwent a rapid lineage diversification. In line with this inference, multi-labeled gene trees predominantly show one of the two homeologs near *D. polytricha* and one near the clade of *D. lutescens* for samples with this placement pattern when two homeologs were recovered (see Supplementary Fig. S26 for an example multi-labeled tree). Given the similar placement patterns across samples from clusters 1–3, we can hypothesize their common origin through hybridization of two diploid lineages, which gave rise to a large clade of >100 exclusively (according to available data) polyploid taxa (Fig. 4).

**Figure 4.**
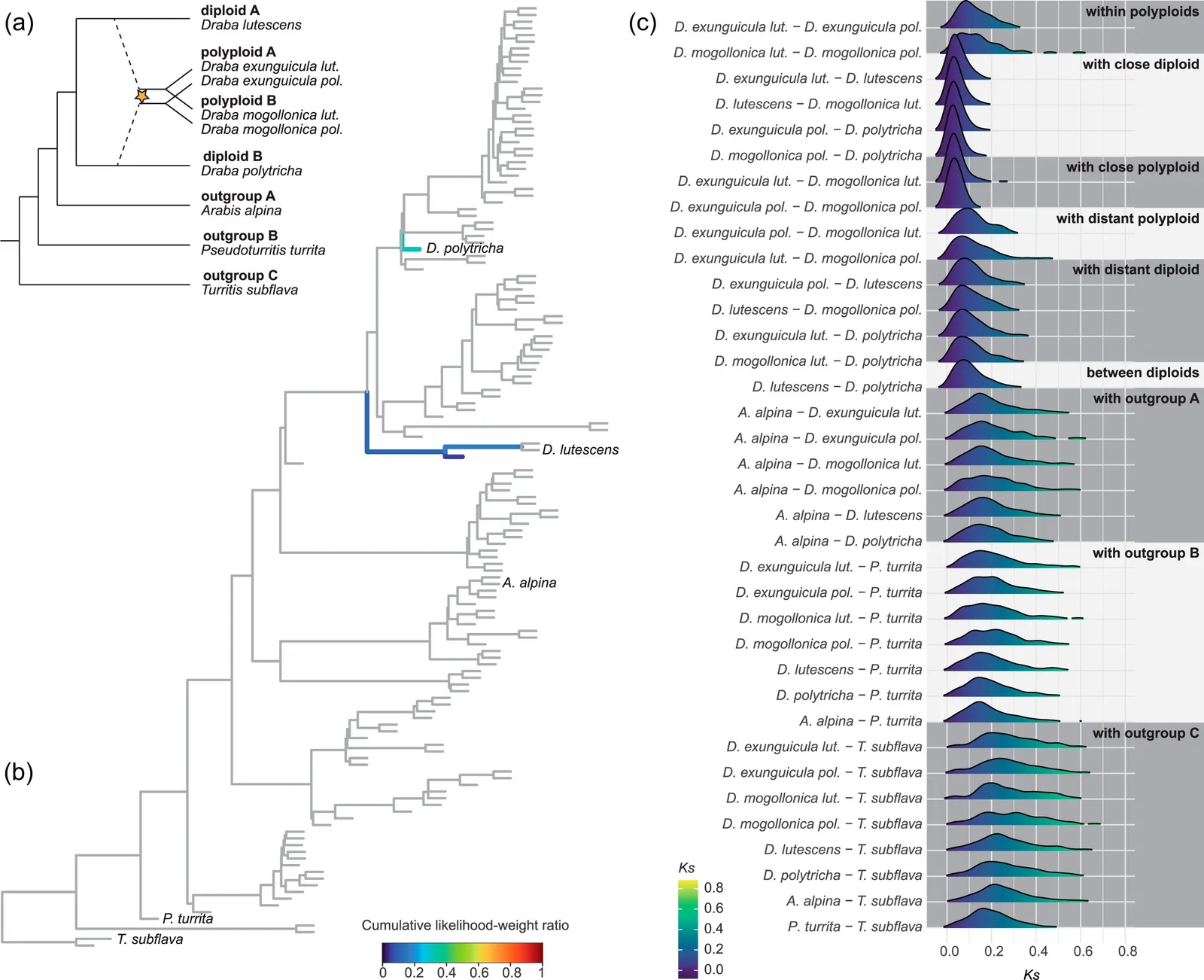
Relative divergence estimation using synonymous substitution rates. Cluster 1 was chosen as an example to illustrate the use of synonymous substitution rates (*Ks*) as a means to support the evolutionary scenario of hybridization inferred from placement patterns and to provide a relative timeline. (a) The evolutionary scenario including the hybrid origin of the polyploid clade from two diploid lineages, along with the taxa analyzed in detail. (b) Placement patterns of cluster 1 into the diploid tree. The cumulative likelihood-weight ratios (lwrs) at branches with lwr > 0.05 are shown, and the diploid taxa selected for detailed analysis of *Ks* are highlighted. (c) Distribution of pairwise *Ks* values between the different species and within polyploids is shown. For polyploids, subsets mapping to the branches leading to diploid A and diploid B, respectively, were analyzed separately.

The second example is clade 30 containing tetraploid *Arabis nordmanniana* and its close relatives *A. mollis* and *A. nepetifolia*. Phylogenetic placement of gene copies suggested a position within the *Arabis alpina* clade, with the ancestor of the polyploids mapping to the long branch separating *A. auriculata* from the other species in the clade. The placement into mainly a single branch seems to suggest autopolyploidization at first glance. However, comparison of within-polyploid *Ks* with pairwise *Ks* between the polyploids and *A. alpina* revealed a lower *Ks* with *A. alpina* (Fig. 5). A possible interpretation of this observation is that the *A. nordmanniana* clade originated through hybridization of an ancestor of the clade around *A. alpina* with a now extinct (or unsampled) ghost lineage. The two homeologs within the tetraploids are then more divergent from each other than one of them from the respective ortholog in *A. alpina*, though both homeologs are placed into the same branch. The gene tree (Supplementary Fig. S26) also shows this pattern. Importantly, pairwise *Ks* values with outgroup taxa were consistent with the respective evolutionary scenarios in both analyzed clusters, and also highly consistent between analyses, indicating their applicability for (relative) time estimates.

**Figure 5.**
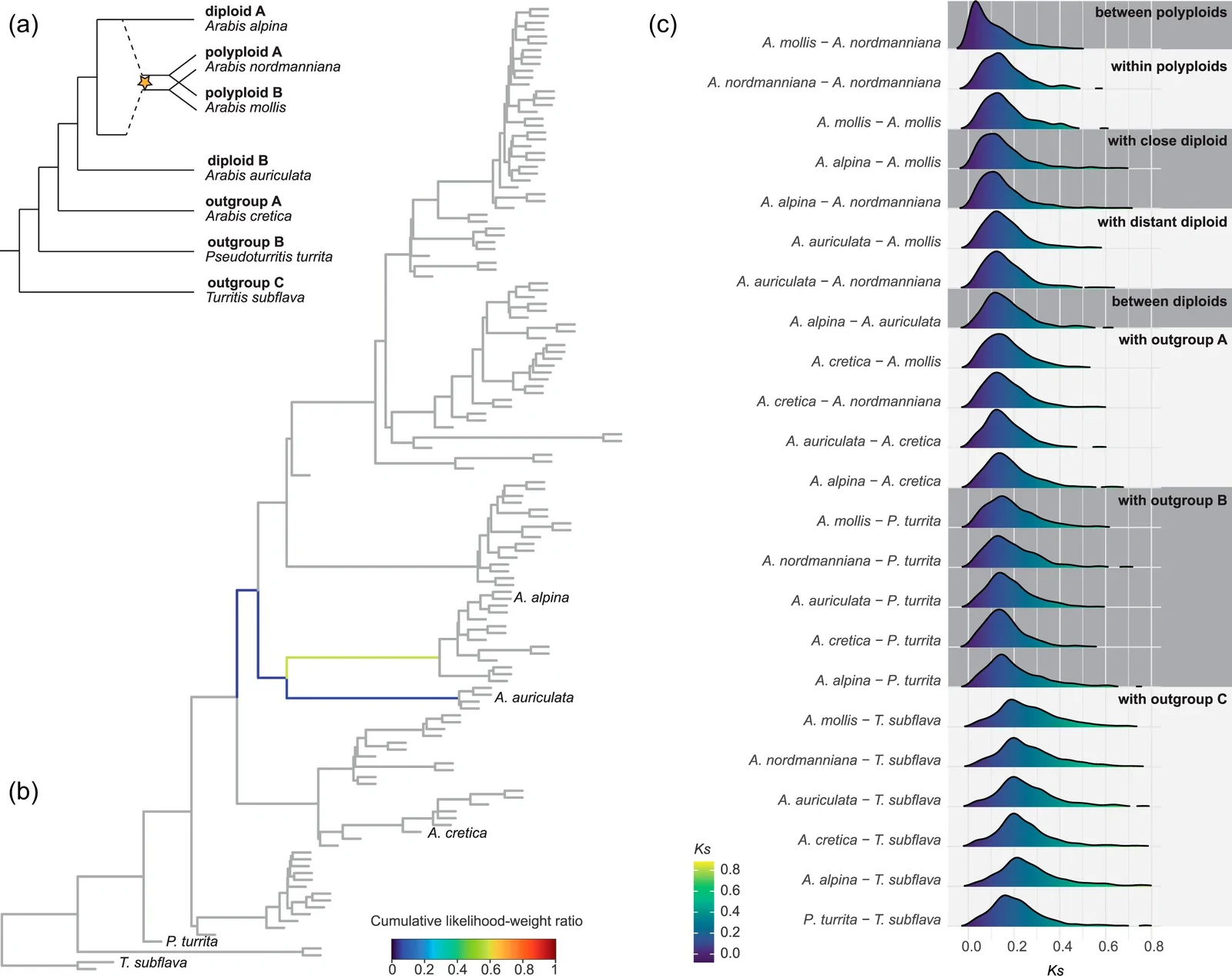
Relative divergence estimation using synonymous substitution rates. Cluster 30 was chosen as an example to illustrate the use of synonymous substitution rates (*Ks*) as a means to support the polyploidization scenario inferred from placement patterns and provide a relative timeline. (a) The evolutionary scenario including the hybrid origin of the polyploid clade from the ancestor of a diploid lineage and an unsampled or extinct ghost lineage, along with the taxa analyzed in detail. (b) Placement patterns of cluster 30 into the diploid tree. The cumulative likelihood-weight ratios (lwrs) at branches with lwr > 0.05 are shown, and the diploid taxa selected for detailed analysis of *Ks* are highlighted. (c) Distribution of pairwise *Ks* values between the different species and within polyploids is shown.

### Identification of Known Polyploidization Events in Arabidopsis

Our test data set from the genus *Arabidopsis* showed results congruent with those of the Arabideae data set with regard to ploidy detection (Fig. 6a). Diploid samples had generally low AD (<2%) and also low LH. Among the diploids, the individuals from the large, outcrossing *A. arenosa* clade showed the highest LH. Autopolyploids from the *A. arenosa* clade had higher AD (>2%), but their LH was not as high as that in allopolyploids (*A. kamchatica* and *A. suecica*) and introgressed tetraploids (*A. lyrata*).

**Figure 6.**
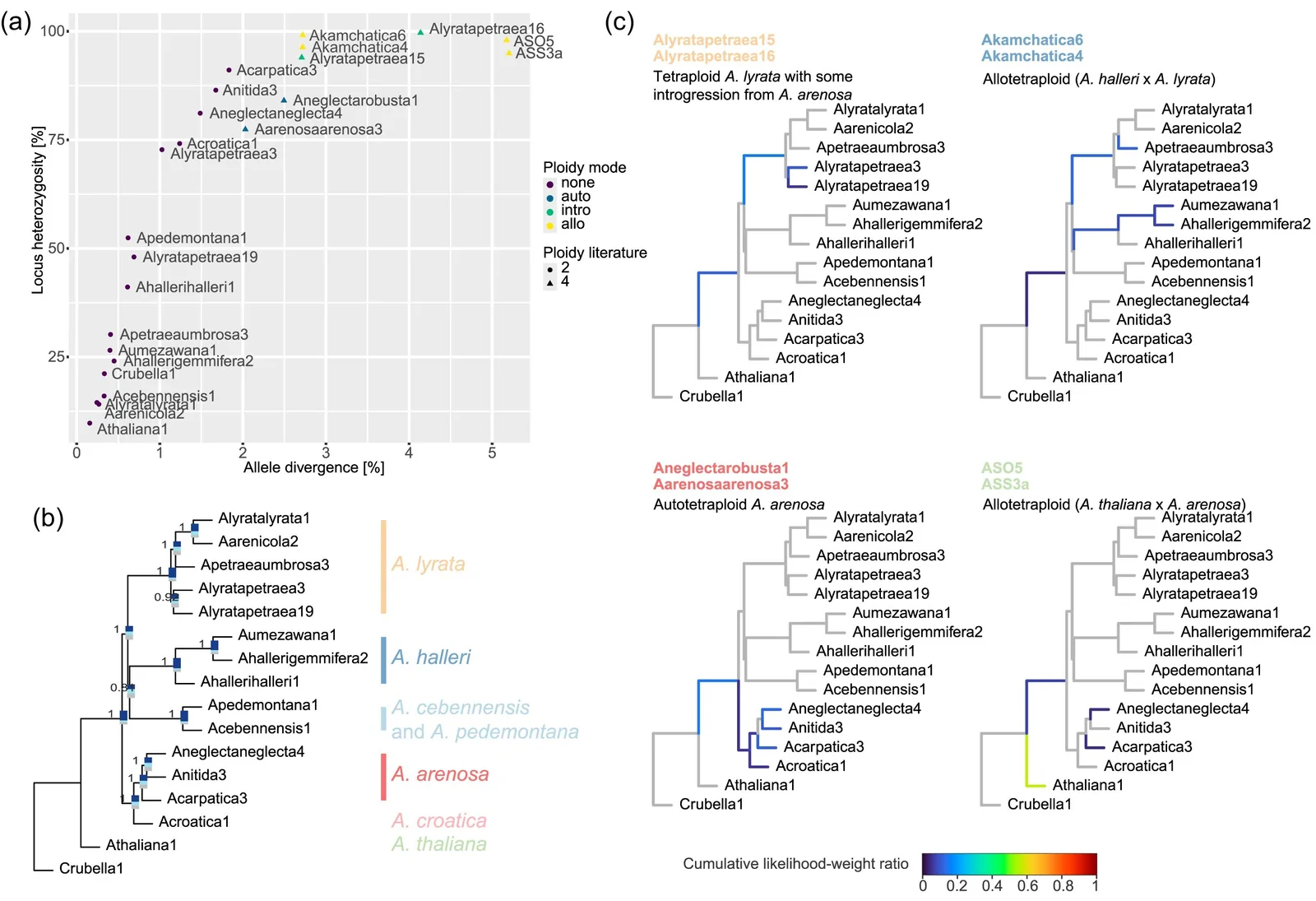
*Arabidopsis* as a test case. (a) Ploidy assessment using allele divergence and locus heterozygosity from HybPhaser in *Arabidopsis*. Mode of polyploidization (autopolyploidization, introgression, allopolyploidization) and known ploidy level (diploid, tetraploid) are indicated. (b) Diploid tree reconstructed using ASTRAL-pro from diploid samples only. Quartet support is shown as node bars, posterior probability as node labels. The major evolutionary lineages are indicated on the right. (c) Phylogenetic placement of homologs into the diploid anchor tree. The eight polyploid samples were grouped into four clusters according to their origin, and placement of the two samples in each cluster is summarized above using a threshold of 0.05.

Phylogenetic reconstruction using all gene copies from diploids recovered the major diploid clades (*A. lyrata, A. halleri, A. cebennensis*, and *A. pedemontana*, and *A. arenosa* with *A. croatica*; Fig. 6b), and inferred a tree topology in line with previous studies (Novikova et al. 2016). Phylogenetic placement of tetraploid samples showed placement patterns as expected from the literature, with ancestry of tetraploid *A. lyrata* in the diploid *A. lyrata* clade, ancestry of allotetraploid *A. kamchatica* in the *A. halleri* and *A. lyrata* clade (notably with affinity to the samples from Asia, where the species originated), ancestry of autotetraploid *A. arenosa* in the *A. arenosa* clade, and ancestry of allotetraploid *A. suecica* in *A. thaliana* and the *A. arenosa* clade.

## Discussion

### Addressing Reticulation and WGD in the Arabideae

The genomics era has produced an enormous and ever-increasing amount of genome-scale data sets leading to the inference of hundreds of recent and ancient WGDs throughout land plant evolution (Vanneste et al. 2014; One Thousand Plant Transcriptomes Initiative 2019; Zuntini et al. 2024). However, current phylogenetic and phylogenomic methods primarily represent evolution as bifurcating trees. The tremendous advances in assembling phylogenomic data sets have exposed the limitations of our current methodology to analyze, classify, and interpret the evolution of plant clades in the face of ancient hybridization and WGD (reviewed in Oxelman et al. 2017; Rothfels 2021; Stull et al. 2023; Joyce et al. 2025). This highlights the urgent need for new approaches that can handle more complex evolutionary scenarios. Recently, Smissen and colleagues (2024) developed an approach to infer the evolutionary history of Australasian *Lepidium*, a taxonomically challenging Brassicaceae genus rich in high polyploids and interploidal and intercontinental hybridization using amplicon sequencing and condensed splits from MUL-networks. Although their data set was much smaller than ours, encompassing only 21 samples and 15 genes, their study illustrates both the demand and the potential for creative solutions to study the origin of allopolyploids in a phylogenetic and systematic context. Similarly, a larger study of the genus *Rosa* used amplicon sequencing and a combination of read-based ploidy estimation, species-tree estimation, and network reconstruction to identify hybrid and polyploid roses and their diploid ancestors (Debray et al. 2022). Similar methodological challenges have been highlighted in phylogenomic studies of reticulate or polyploid groups in several other clades, such as *Lachemilla* (Morales-Briones et al. 2021), polyploid fern lineages (Rothfels et al. 2017; Tseng et al. 2024), and bryophytes (Patel et al. 2023).

Our study system, the Brassicaceae tribe Arabideae, exhibits several of the same challenges commonly faced by current phylogenomic data sets: diverse levels of polyploidy, with ploidy levels ranging up to hexadecaploid, unknown ploidy status in taxa only available as herbarium vouchers, a large evolutionary lineage with diversification into over 500 taxa in under 20 Ma, and an unknown number of (nested) WGD events. In this study, we aimed to develop an approach to investigate the evolutionary history of such a clade using target enrichment data. The outputs at the specific stages of our analysis pipeline are summarized in Figure 7.

**Figure 7.**
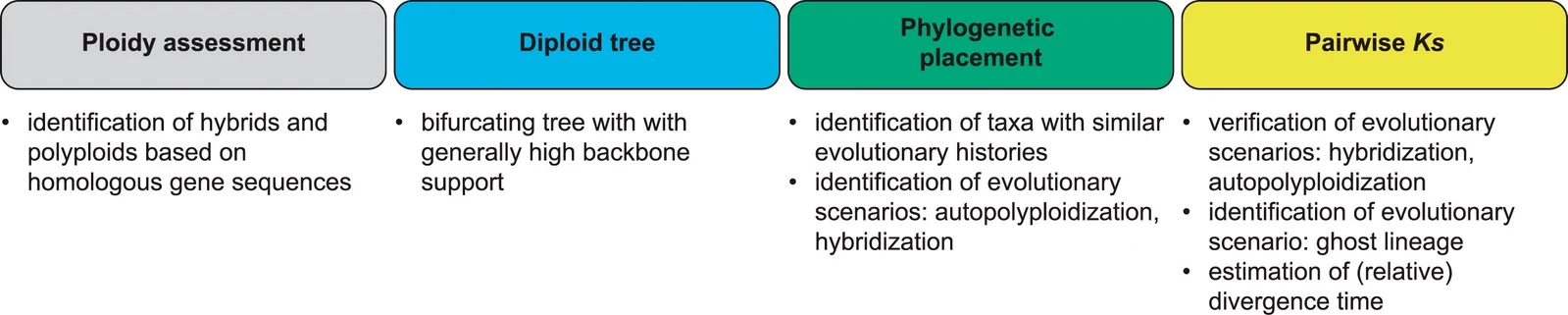
Output summary. The output of the different parts of our analysis pipeline is summarized above. Identification of hybrids and polyploids is the prerequisite for reconstructing a diploid tree, which provides a bifurcating phylogeny with generally high backbone support. Phylogenetic placement of homologs furthermore allows us to identify hybrid or polyploid samples with a similar evolutionary history and at the same time distinguish between evolutionary scenarios involving autopolyploidization and hybridization. Finally, pairwise synonymous substitution rate (*Ks*) can be used as further evidence for these evolutionary scenarios, identify those involving hybridization with a ghost lineage, and provide a (relative) estimate of divergence time.

### Incorporating Homeologs and Unknown Ploidy in Phylogenomic Analyses

Target enrichment has become widely used across a diverse set of taxa, often employing the same targeted loci through universal probe sets (Johnson et al. 2019; Bartoš et al. 2023), which allows for the combination or extension of data sets in future studies. This also predisposes target enrichment phylogenomics data sets to follow-up analyses using newly developed approaches. The prevalent focus on single-copy genes currently often leads to filtering of phylogenomics data sets with the aim of excluding duplicated gene sequences (e.g., Hendriks et al. 2023; Zuntini et al. 2024), so that only subsets of the available data are finally analyzed. However, we argue that homeologs originating from WGDs, whether through autopolyploidization or hybridization, can and should be used to gain a deeper understanding of plant evolutionary history.

Many phylogenomic studies a priori select taxa that are assumed to be diploid (e.g., Hay et al. 2023), include only few allopolyploids, or only those with relatively low ploidy level rarely exceeding tetraploid, so that utilizing existing bioinformatics pipelines for assembling multiple gene copies from such taxa is feasible (Nauheimer et al. 2021; Freyman et al. 2023; Patel et al. 2023). However, the ploidy status of many plant species is still unknown (Halabi et al. 2023) and cannot easily be determined from material available as herbarium vouchers or dried leaf material, especially older material (Viruel et al. 2019). Although allele frequency-based approaches have been used to estimate ploidy levels from target enrichment data (Viruel et al. 2019; Gaynor et al. 2024), these methods may be limited to ploidy levels up to tetraploid (Weiß et al. 2018) or hexaploid (Gaynor et al. 2024) as they explicitly model allele frequency distributions under fixed ploidy hypotheses. Although our study does not estimate exact ploidy levels but rather distinguishes diploid from polyploid samples, the presence of taxa with higher ploidy levels makes model-based approaches inappropriate for our data set. Other studies have used the number of paralog warnings issued by HybPiper as a means to identify polyploids; however, this approach was less successful in samples with lower coverage (Patel et al. 2023). We detected high numbers of genes with paralog warnings even in samples with lower coverage (Supplementary Fig. S5), and also in some samples from diploid taxa. Instead of the paralog warnings approach, we therefore employed a sequence-based approach that utilizes the divergence and diversity of target enrichment loci to select diploids. Ploidy was unknown for 44% of our Arabideae samples, but the high consistency between predicted and known ploidy status for the remaining samples indicated a high reliability of the method. Unlike chromosome counts, the sequence-based approach can detect taxa with past hybridization events that have undergone diploidization, reverting their chromosome count to diploid level. Conversely, homoploid hybrids are likely detected as “polyploids” using our approach, due to their high heterozygosity. However, such sequence-based approaches could also falsely identify polyploids in case of contamination. Although low-level contamination with a small fraction of reads from another sample should be below the detection threshold for heterozygous sites, high levels of contamination, such as a “mixed” sample with approximately equal parts from two diploids, would likely be detected as polyploids with this method as well as others. On the other hand, our method may overlook recent autopolyploids due to its reliance on allele divergence. This oversight appears to be minor in our data set, as we identified only four samples where chromosome numbers reported in the literature suggest polyploidy, yet the respective sample was classified as “diploid.” A high reliability of the method was also indicated by the successful identification of all known polyploids in the genus *Arabidopsis* (Fig. 6), where in particular the allopolyploids were easily identifiable using locus heterozygosity and allele divergence, but also autotetraploids showed high levels of allele divergence.

### Challenges of Homeolog Phasing and Our Alternative Framework

Another key challenge when working with potential allopolyploids is phasing loci into the respective subgenomes. Over the past few years, several bioinformatics pipelines have been developed to phase homeologs into subgenomes. For example, Leal et al. (2023) employed genomic polarization of homeologs in allopolyploids, which was successfully tested in *Arabidopsis* and particularly effective in allopolyploid *A. suecica*, where the parental genomes of *A. arenosa* and *A. thaliana* were highly divergent. Although scalable to a large number of loci and samples, genomic polarization of homeologs is currently limited to allotetraploids. Another approach, homologizer (Freyman et al. 2023), uses a tree-based method to phase homeologs and can handle higher ploidy levels. However, some implementations of this software for complex data sets can be computationally demanding. A widely used approach for phasing homeologs and removing potential paralogs is HybPhaser (Nauheimer et al. 2021). In addition to assessing heterozygosity of the sequenced loci as we have done in this study, this software can be used to phase homeologs, but requires a priori selection of reference taxa. In contrast, our approach of phylogenetic placement of gene copies does not phase loci into subgenomes. Instead, it provides a visual representation of summarized gene trees, from which the reticulate evolutionary history of the studied species can be deduced. Our method is applicable to various ploidy levels, can accommodate nested hybridization events as well as diploidized polyploids, and can be scaled to large data sets with hundreds or thousands of loci and samples. It also does not require the homeologs to be retained and is therefore also suitable for species of allopolyploid origin after large parts of the diploidization process have been completed, because the phylogenetic placement approach does not require any gene tree to have multiple tips for a polyploid taxon; placement into two different branches across many genes is sufficient to infer the hybrid origin. As such, it serves as a valuable first step for identifying samples that can serve as examples for studying particular reticulation events in further detail, or those that can serve as references for approaches like HybPhaser.

### A Scalable Framework for Complex Evolutionary Histories

Even after successful assembly and identification of orthologs and homeologs, another challenge arises in the next step: phylogenetic reconstruction. Traditional bifurcating trees, commonly used in phylogenetics, are inherently unable to depict reticulate evolution. Although network reconstruction offers an alternative (Solís-Lemus and Ané 2016; Solís-Lemus et al. 2017), it can be computationally intensive and often impractical. Phylogenomic studies of clades with complex evolutionary histories therefore often employ a multi-step approach, starting with species tree inference and followed by detailed investigation of particularly interesting taxa in smaller data sets. This method is frequently justified by cytonuclear incongruence or uniform taxon sampling across clades (e.g., Morales-Briones et al. 2021; Debray et al. 2022; Fu et al. 2023). Although our approach of phylogenetic placement also uses successive analyses (Fig. 1), it offers a unique advantage by allowing all data to be viewed simultaneously and could be scaled to even larger data sets. This enables the assessment of high numbers of included diploid taxa (and their ancestral branches) as potential origins of polyploid and hybrid taxa, providing an informative basis for subsequent selection of subsets for further analyses. For example, this approach could facilitate network analyses that encompass all lineages involved in the origin of hybrid taxa, and it can also be used to inform sample selection for comparative genomics projects. Additionally, it is suitable for evolutionary scenarios that involve multiple nested WGDs, an aspect often overlooked in phylogenomic studies.

### Ks-Based Insights into Polyploidization and Ghost Lineages

Phylogenomic reconstructions from diploid samples in our data set using conventional tree-building methods resulted in bifurcating trees largely consistent with previous single-marker phylogenetic studies (Jordon-Thaden et al. 2010; Karl and Koch 2013), which displayed high support values (local posterior probability and quartet support ) in the backbone of Arabideae. Additionally, analyzing synonymous substitution rate (*Ks*) between gene copies provided valuable insights not only into the placement of polyploid clades and the potential parents of allopolyploids, but also their relative times of origin. Within-species *Ks* is commonly used in comparative genomics studies—particularly when whole-genome sequences are available—to infer the timing of WGDs across taxa (e.g., Hoang et al. 2023). It is also used in conjunction with between-species *Ks* to provide a relative timing context among related clades (Hoang et al. 2024). *Ks* has previously been employed in phylogenetic studies to assess the number of WGDs and compare patterns of (shared) *Ks* peak distribution based on whole-genome and transcriptome data (Morales-Briones et al. 2021). In our study, we calculated pairwise *Ks* between samples and within polyploids to evaluate the presence of past WGDs, as well as patterns and order of divergence. We were able to assess *Ks* within and between all samples from target enrichment data directly and infer three modes of polyploidization: hybridization (allopolyploidization), autopolyploidization, as well as hidden allopolyploidization involving a ghost lineage. The latter, in particular, only became apparent through the interpretation of within- and between-species *Ks* peaks. Ghost lineages can be difficult to discern using phylogenetic methods and thus are largely overlooked. Some phylogenetic methods using multi-labeled trees are able to infer ghost lineages based on gene trees (e.g., Jones et al. 2013; Freyman et al. 2023; Patel et al. 2023), and recently, methods derived from population genetics, such as coalescent modeling or comparative genomics, have been employed to hypothesize or detect ghost lineages in specific scenarios (Luo et al. 2017; Ru et al. 2018; Dong et al. 2022; Sancho et al. 2022; Chang et al. 2023). Network analyses have also been used to reconstruct evolutionary scenarios involving ghost lineages as the parents of even multiple hybrid samples in *Erysimum* (Osuna-Mascaró et al. 2023). These studies altogether underscore the significant role that ghost lineages can play in the evolutionary history of various taxa. The use of *Ks* for discovering such ghost lineages adds another layer of evidence to support such events and could be a valuable tool in other studies.

### Conclusions and Future Direction

In summation, there is no one-size-fits-all approach to phylogenomics of hybrids and polyploids; different data sets may require different strategies. For instance, when genome sequences are available, subgenome analysis may be a viable option (Guo et al. 2021). Our approach offers first insights into some of the evolutionary processes that have shaped phylogenetically complex and highly polyploid clades using target enrichment data (and could easily be extended to other multi-locus data sets). These initial insights can and should serve as a foundation for further and more focused studies, for example on genome-scale, to develop a more comprehensive understanding of the intricate patterns of reticulate evolution found in plants. Moreover, our results highlight the necessity for developing methods to study trait and character evolution in the context of hybridization and polyploidization (Wang et al. 2021). Bifurcating trees fail to capture the full evolutionary reality of such clades, and their evolutionary potential may be influenced by contributions from multiple lineages. We strongly advocate for a more cautious approach: carefully examining the sequencing data and employing methods that can effectively untangle these complex evolutionary histories. Oversimplification into phylogenetic trees can significantly limit the conclusions drawn from comparative phylogenetic or biogeographic studies. Therefore, it will be essential to adopt methodologies that account for the complexity and reticulate nature of plant evolution to fully understand their evolutionary history.

## Supplementary Material

syag007_Supplemental_Files

## Data Availability

Raw sequencing reads generated for this study are available at ENA (https://www.ebi.ac.uk/ena/browser/home) under accession PRJEB72669. Supplementary materials, including data files and code/scripts necessary to reproduce the analyses, can be found in the Dryad data repository (https://doi.org/10.5061/dryad.xksn02vqn). The formal description of the new taxon *Turritis subflava* has been presented with Supplementary Text.
