# Supplementary material for: Unravelling Complex Hybrid and Polyploid Evolutionary Relationships Using Phylogenetic Placement of Homologous Gene Copies from Target Enrichment Data
Source: Syst Biol. 2026 Jan 27;75(6):1120–39. doi: 10.1093/sysbio/syag007 (PMC13637605; doi:10.1093/sysbio/syag007)
Supplement: Supplementary file 1 [file syag007_supplemental_files.zip › Arabideae_WGDs_Supplements_revision2.pdf]

Supplementary Material

Unravelling complex hybrid and polyploid evolutionary relationships using phylogenetic placement of homologous gene copies from target enrichment data

Nora Walden[1] , Christiane Kiefer[1] , Marcus A. Koch[1]

[1]*Centre for Organismal Studies, Heidelberg University, Im Neuenheimer Feld 345, 69120 Heidelberg, Germany*

Corresponding authors:

Nora Walden, nora.walden@cos.uni-heidelberg.de

Marcus Koch, marcus.koch@cos.uni-heidelberg.de

Table of contents

Supplementary Text 3

New taxonomic combination: *Turritis subflava* 3

Supplementary Figures 5

Figure S1. Mapping rate. 5

Figure S2. Gene recovery rate. 6

Figure S3. Gene selection. 7

Figure S5. Number of paralog warnings. 9

Figure S6. Detection of multiple gene copies by ploidy. 10

Figure S7. ASTRAL-pro tree based on all gene copies from diploid samples. 11

Figure S8. Support values for ASTRAL-pro tree based on all gene copies from diploid samples. 12

Figure S9. Phylogenetic placement 13

Figure S10. Scatterplot of clustering height and number of clusters. 14

Figure S11. Dendrogram of hierarchical clustering. 15

Figure S12. Dendrograms for gene copy placement of clusters 1-4. 16

Figure S13. Dendrograms for gene copy placement of clusters 5-8. 17

Figure S14. Dendrograms for gene copy placement of clusters 9-12. 18

Figure S15. Dendrograms for gene copy placement of clusters 13-16. 19

Figure S16. Dendrograms for gene copy placement of clusters 17-20. 20

Figure S17. Dendrograms for gene copy placement of clusters 21-24. 21

Figure S18. Dendrograms for gene copy placement of clusters 25-28. 22

Figure S19. Dendrograms for gene copy placement of clusters 29-32. 23

Figure S20. Dendrograms for gene copy placement of clusters 33-36. 24

Figure S21. Dendrograms for gene copy placement of clusters 37-40. 25

Figure S22. Dendrograms for gene copy placement of clusters 41-43. 26

Figure S23. ASTRAL-pro tree based on individual exon copies from diploid samples. 27

Figure S24. Phylogenetic placement for exon sequences. 28

Figure S25. Three evolutionary scenarios for the emergence of polyploids analyzed with *Ks*. 29

Figure S26. Multi-labeled gene tree. 30

Supplementary Text

New taxonomic combination: *Turritis subflava*

Turritis subflava (B.M.G. Jones) M. Koch, D.A. German & R. Karl comb. nov.

≡ *Arabis ochroleuca* Boiss. & Heldr. in Boissier, **Diagn. Pl. Orient., ser. 2, 1: 21 (1854),** *nom. illeg.* (non *Arabis ochroleuca* Lam., Encycl. méth. Bot. 1(1): 218. 1783).

≡ *Arabis subflava* B.M.G. Jones; in **Feddes Repert. Spec. Nov. Regni Veg. 69: 60 (1964).**

Lectotype (designated by Strid & Titz, 1986: 267):—[Greece] in m. Kyllenes, reg. alpina 6500', 6 July 1848, T.H.H. von Heldreich 2161 (G-BOIS: G00332068; isolectotypes: B100201869, WU 0067983).

Further specimens inspected:

KEW (K) K000693238, coll.: Orphanides T.G., #247; 28th June 1852; Montis Ziriae supra Liviadia (Greece)

VIENNA (W) 2007-0000061, coll.: Walter J., #7895, 24th June 1996; Cyprus, Trodos mountains

HEIDELBERG (HEID) HEID505730, HEID505731, HEID505739, HEID505740, coll. Marcus A. Koch, #19-00677, 19th April 2019; Cyprus, Mt. Olympos, Trodos mts., 1,750 m a.s.l.;

Distribution

Turritis subflava is distributed in mountaneous regions in Southeastern Greece and Aegean Islands. The perennial species occurs between 1,500 m and 2,200 m a.s.l. in open habitat (screes, grassland) and rocky areas such as inland cliffs and mountain peaks). The species is considered to be threatened (Bachman et al. 2024).

Phylogenetic evidence and morphology

Turritis subflava has been studied in detail by Karl & Koch (unpublished) as *Arabis subflava* and two accessions (RK145: Berlin - B100201867; and A77M: Munich - M21427) have been analysed for DNA sequence variation of the nuclear encoded Internal Transcribed Spacer 1 and 2 of nuclear encoded ribosomal DNA and plastid encoded *trn*L-*trn*LF intergenic/genic region. DNA sequences can be found in GenBank (Karl 2012).

The species clearly groups within the genus *Turritis*, which is assigned as the only genus to tribe Turritideae, and which consists of two additional accepted species only: *T. glabra* and *T. laxa* (*BrassiBase*: https://brassibase.cos.uni-heidelberg.de/).

The genus *Turritis* is morphologically very similar to genus *Arabis* (still paraphyletic within tribe Arabideae). *Turritis subflava* is very close to the other two members of this genus *Turritis* sharing with them indumentum of predominantly 2-4-rayed stalked trichomes confined to the lower part of plant, being glabrous and glaucous above, having deeply amplexicaul cauline leaves and usually creamy petals.

Siliques in *T. subflava* are not fully compressed as in *Arabis* spp., but somewhat tetragonal-latiseptate (rhomboid in cross section). Seeds are uniseriate and only very slightly biseriate (vouchers at HEID).

References

Bachman, S.P., Brown, M.J.M., Leão, T.C.C., Nic Lughadha, E. and Walker, B.E. (2024), Extinction risk predictions for the world's flowering plants to support their conservation. New Phytol, 242: 797-808. https://doi.org/10.1111/nph.19592

Strid, A. & Titz, W. (1986) *Arabis* L. *In:* Strid, A. (Ed.) *Mountain flora of Greece*. Vol. 1. Cambridge University Press, Cambridge, pp. 261–268.

Jones, B.M.G. (1964) *Arabis subflava* B. M. G. Jones, nom. nov. *In:* Heywood, V.H. (Ed.) Flora Europaea Notulae Systematicae ad Floram Europaeam spectantes No 3. *Feddes Repertorium Specierum Novarum Regni Vegetabilis; Zeitschrift für taxonomische und chorologische Botanik* 69 (1): 60–61.

Karl R. (2012) Phylogeny and systematics of the tribe Arabideae with a special focus on the genus *Arabis* L. PhD thesis. Heidelberg University. Germany. pp. 1-154.

Supplementary Figures

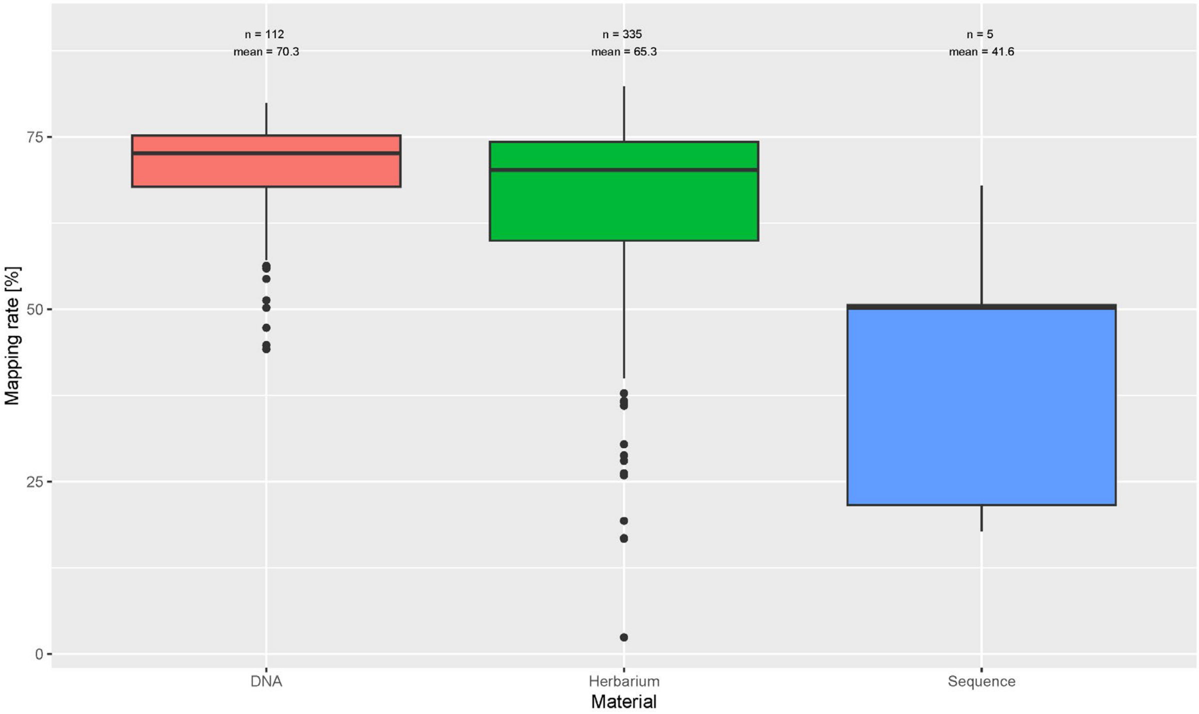

Figure S1. Mapping rate. Percentage of reads mapped to the target reference is given for each source material. New sequences were generated for this study either from DNA used in previous studies or from DNA extracted from herbarium vouchers. Additionally, sequences from five samples from ENA/GenBank were added.

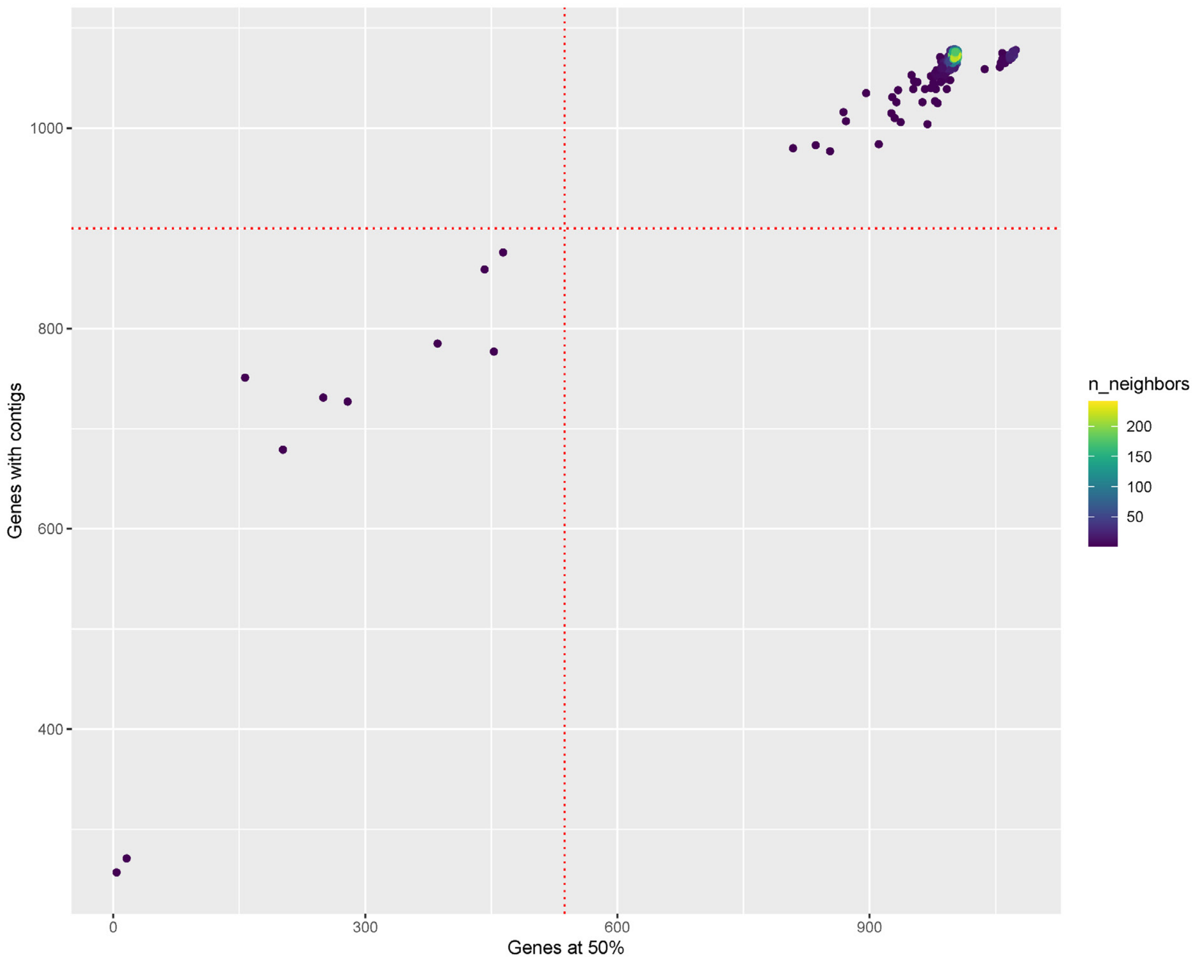

Figure S2. Gene recovery rate. Point density plot for the number of genes with contigs and number of genes covered over a minimum length of 50% of the target reference. Only samples with at least 900 genes with contigs and half of the total genes covered at 50% were retained for further analysis; the selection threshold is indicated by red dotted lines. The final dataset thus comprised 442 samples.

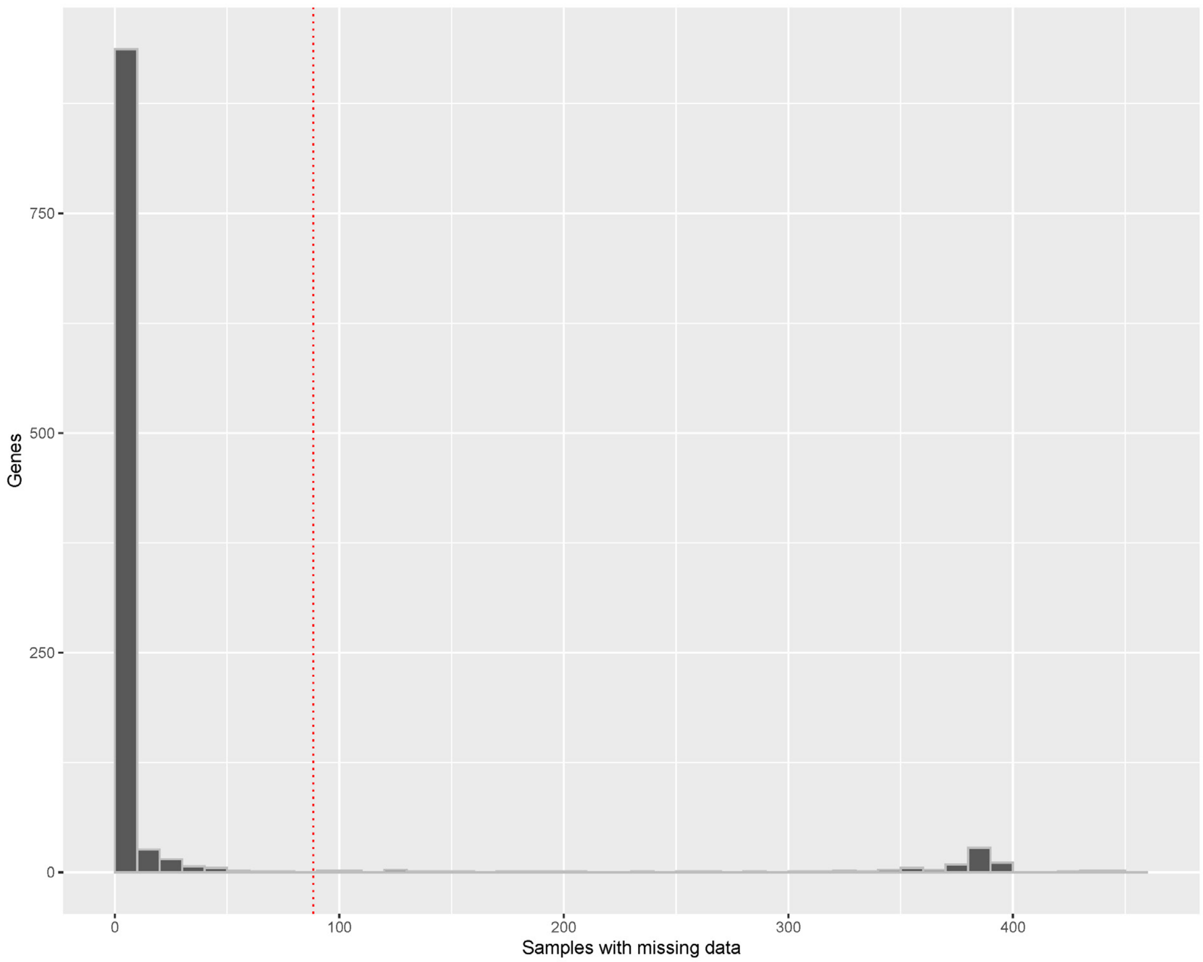

Figure S3. Gene selection. Histogram of number of genes and samples with missing data. Only genes with less than 20% of samples missing were retained for further analysis; the selection threshold is indicated by red dotted lines. The final dataset thus comprised 994 genes.

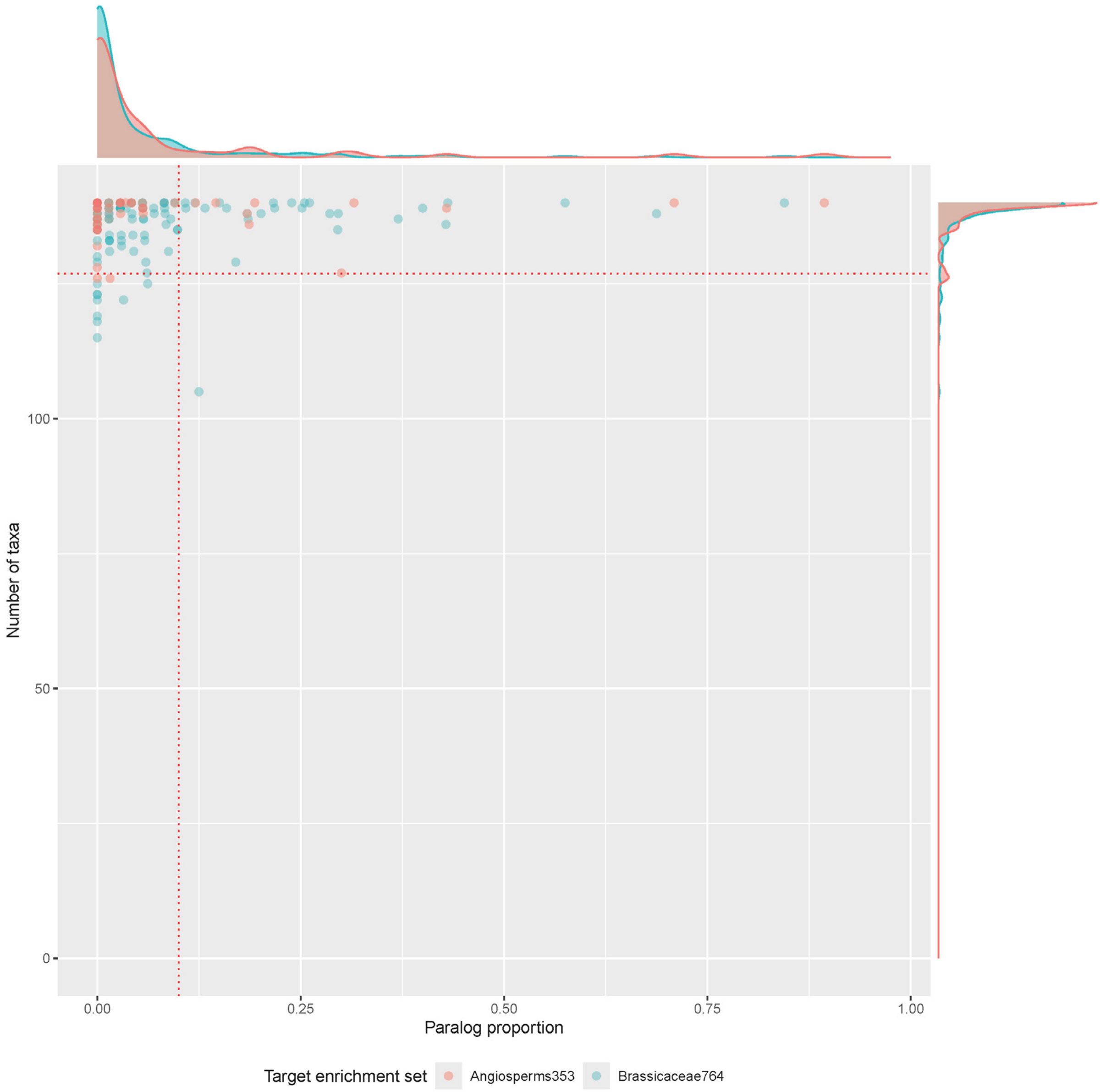

Figure S4. Gene selection for phylogenetic placement. Proportion of samples with multiple copies and number of taxa (of 138) is shown. Only genes with less than 10% of samples with multiple copies and at least 90% of samples present were used.

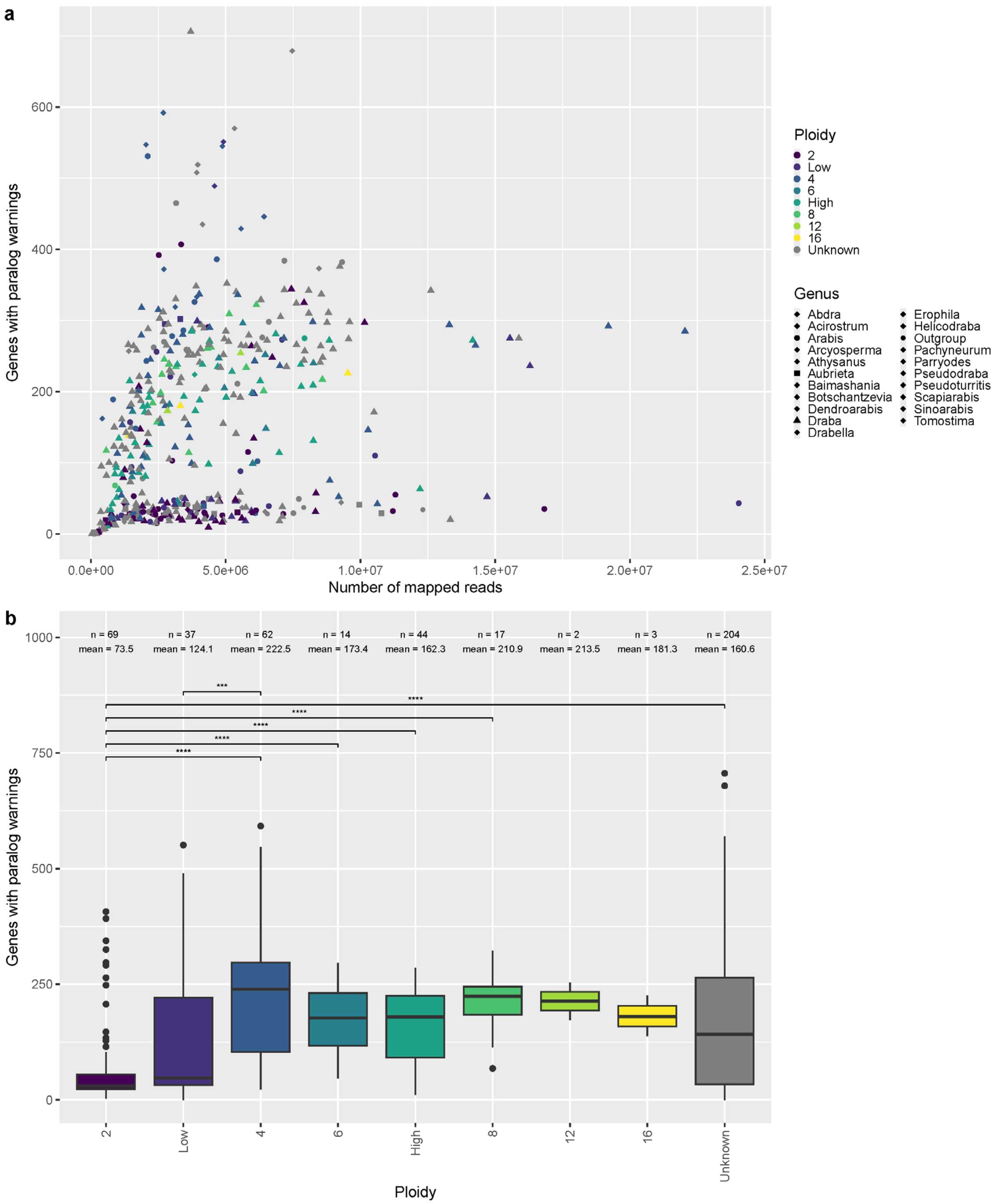

Figure S5. Number of paralog warnings. (a) Scatterplot of number of genes with HybPiper paralog warnings and number of mapped reads. The larger genera are indicated with symbols, ploidy levels as reported in the literature using the color scale. (b) Boxplot of number of genes with paralog warnings by ploidy level reported in the literature. A Kruskal-Wallis test revealed significant differences between groups (Kruskal-Wallis chi-squared = 62.163, df = 8, p-value = 1.751e-10); only significant pairwise Wilcoxon rank-sum test are shown (***: $p \le 0.001$; ****: $p \le 0.0001$).

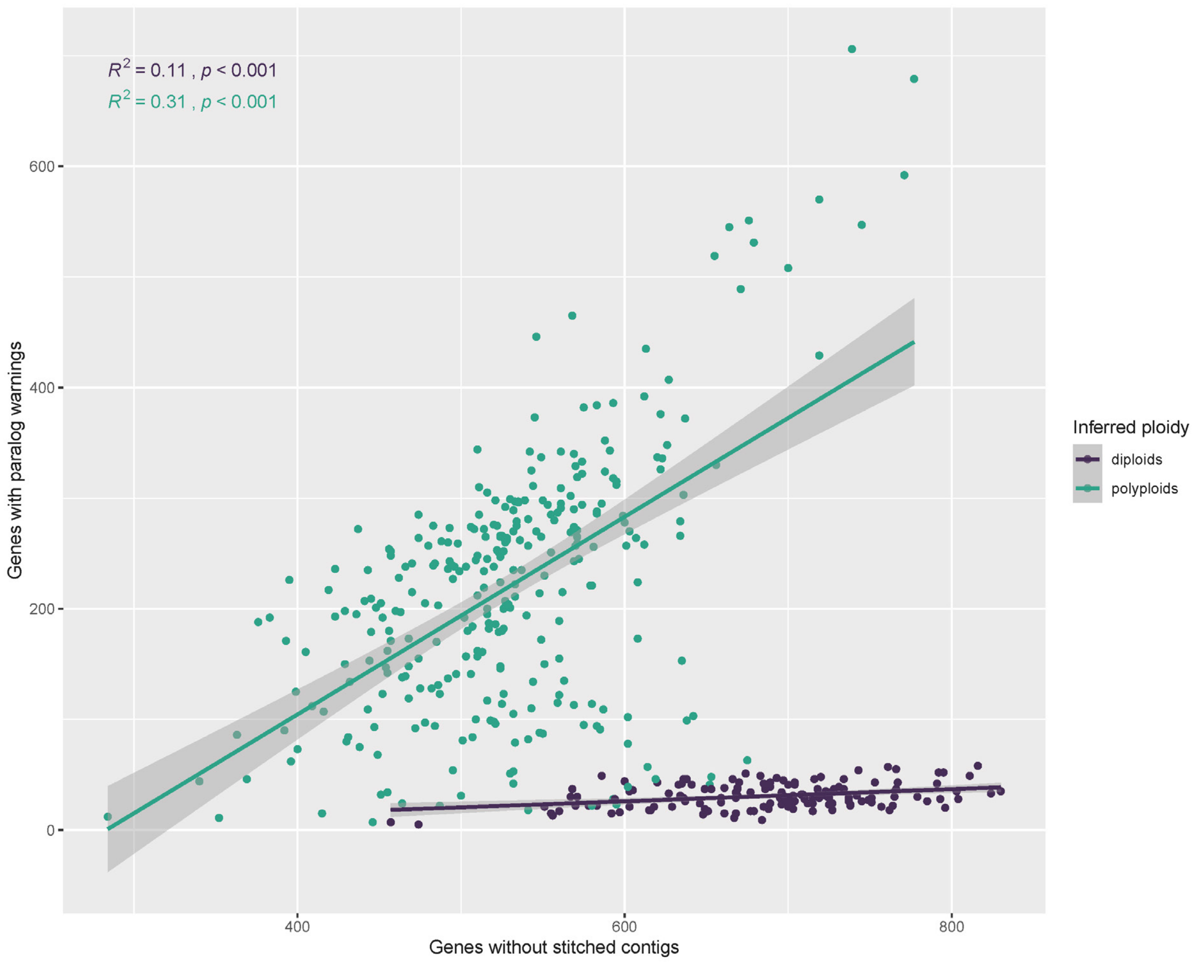

Figure S6. Detection of multiple gene copies by ploidy. Scatterplot of the number of genes with paralog warnings from HybPiper and the number of genes without stitched contigs (i.e. those where gene sequences could be assembled as a single contig in SPAdes).

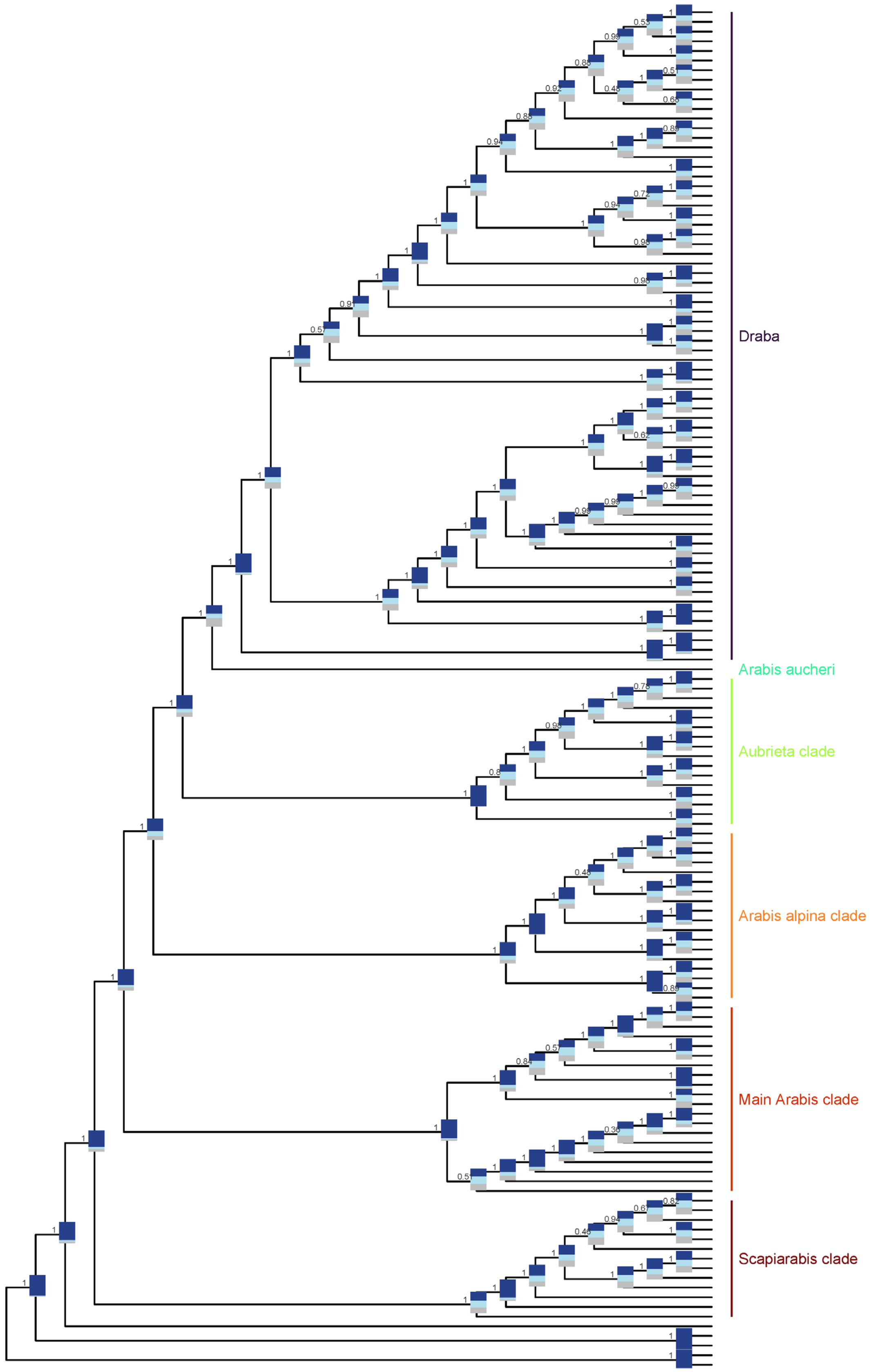

Figure S7. ASTRAL-pro tree based on all gene copies from diploid samples. The tree was reconstructed from 994 gene trees that included all gene copies assembled by HybPiper. Posterior probabilities for the first topology are shown as node labels and quartet scores are shown as bar charts: dark blue for the first quartet, light blue for the first alternative.

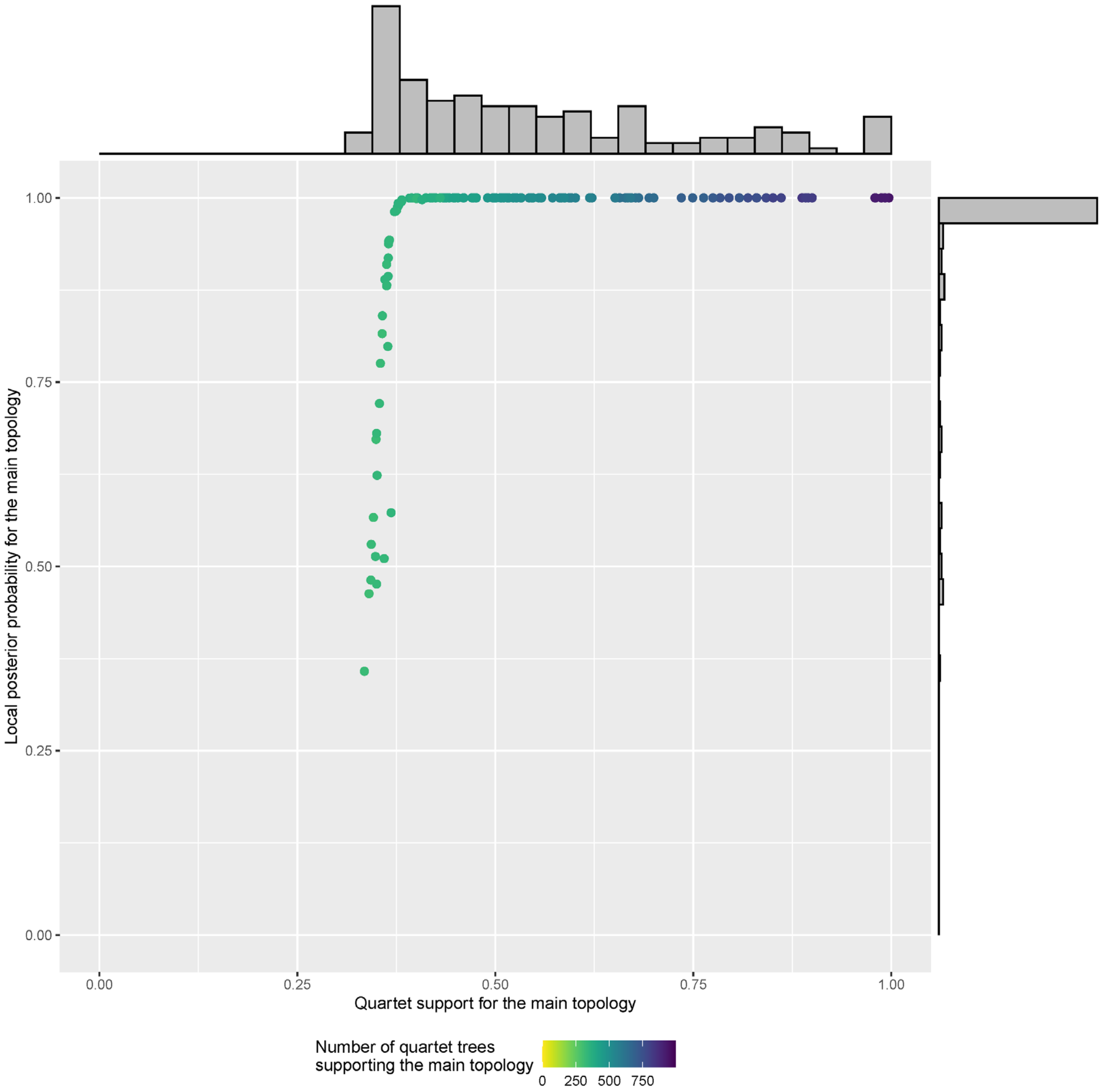

Figure S8. Support values for ASTRAL-pro tree based on all gene copies from diploid samples. Posterior probability and quartet support at each branch are shown for the first topology; color scale shows the number of quartet trees in all the gene trees that support the first topology relative to the total number of genes (994).

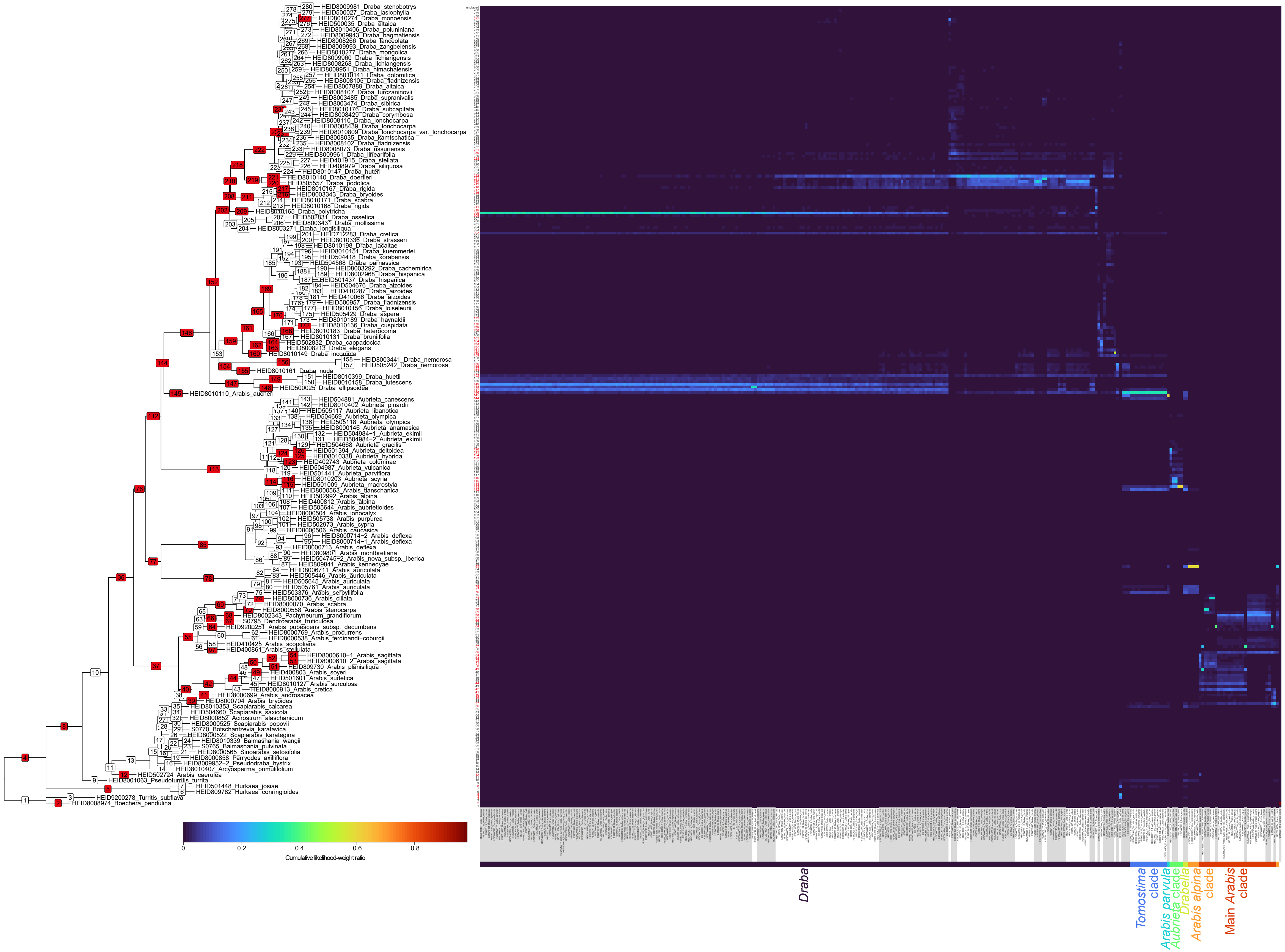

Figure S9. Phylogenetic placement. Cumulative likelihood-weight ratios (lwr) over all gene copies from ‘polyploid’ samples are given. Samples are arranged by cluster on the x-axis, and edges in the tree are displayed on the y-axis. The dendrogram of the diploid tree was added for orientation; edges with mean lwr ≥ 0.05 in at least one cluster are highlighted in red.

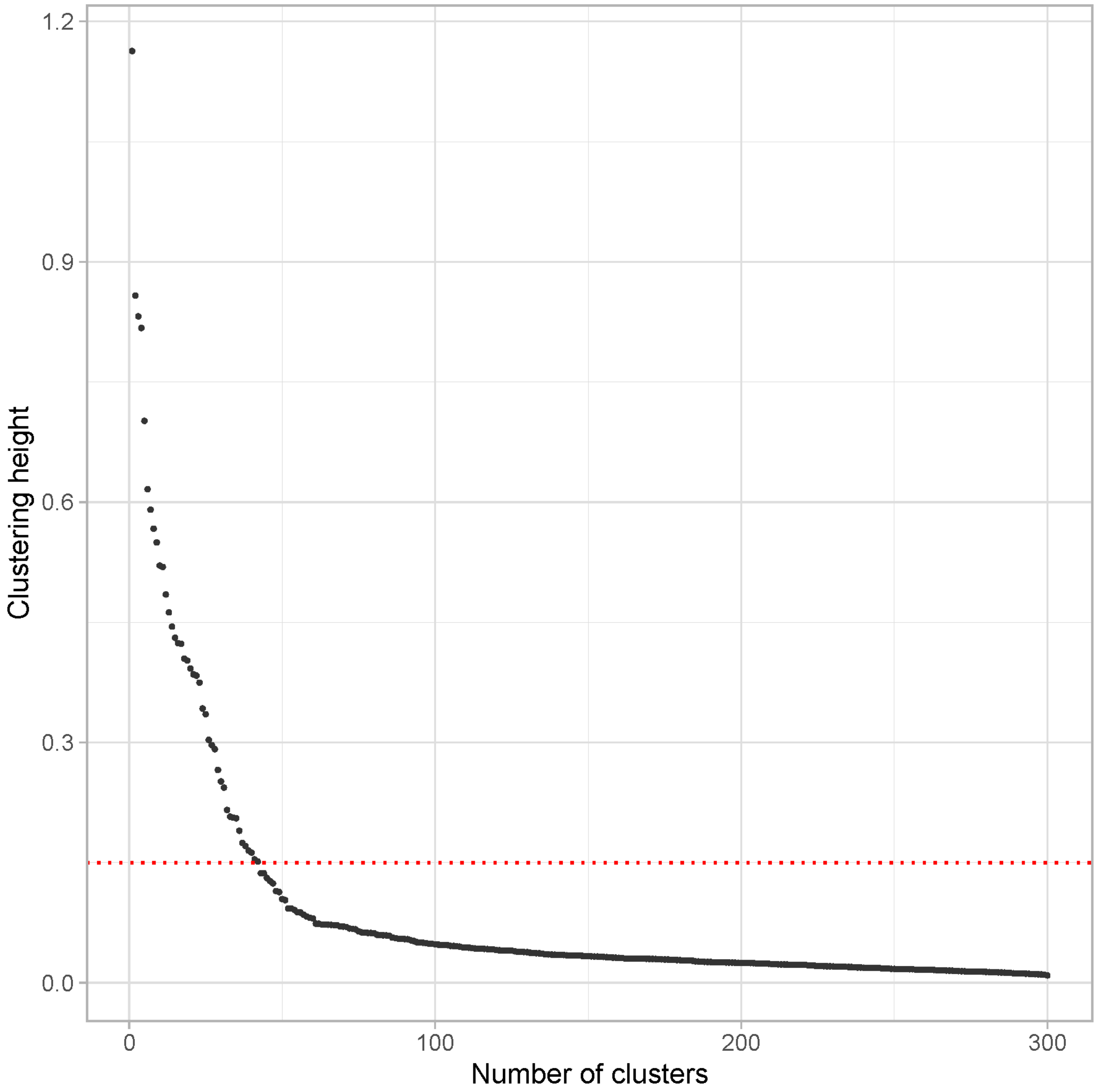

Figure S10. Scatterplot of clustering height and number of clusters. Distribution of clustering heights after hierarchical clustering of distance between normalized paralog placement assignment. A threshold of 0.15 was chosen to select clusters and is indicated by the red dashed line.

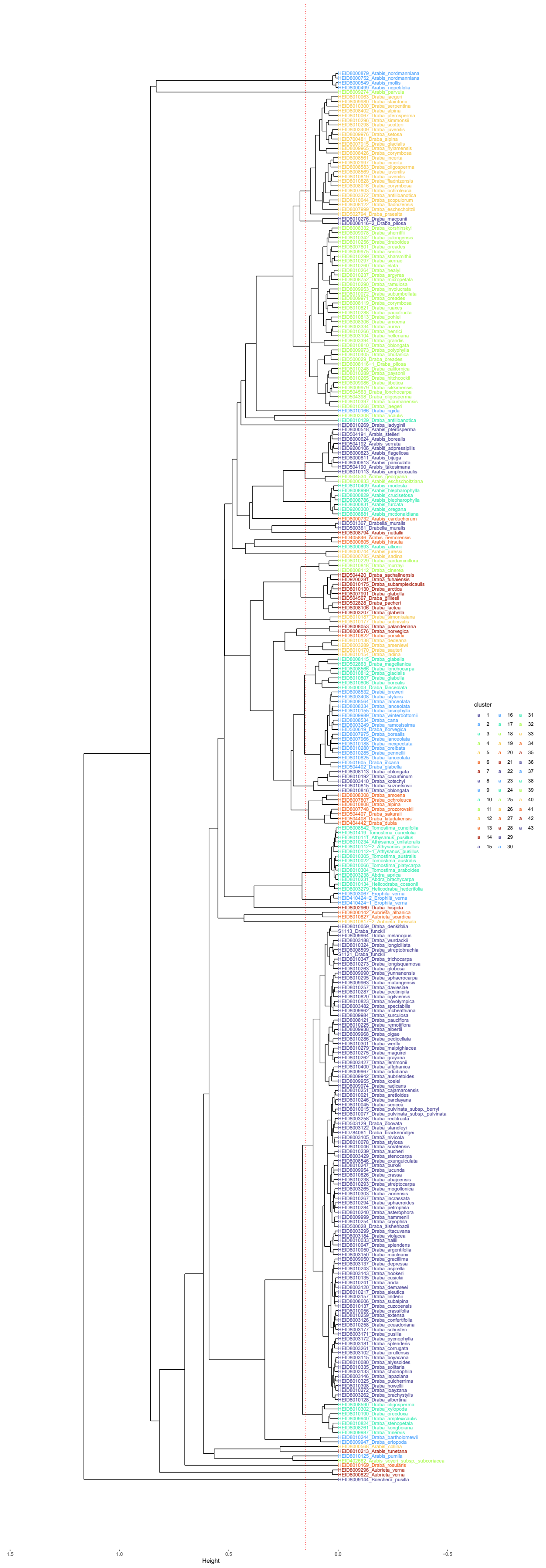

Figure S11. Dendrogram of hierarchical clustering. The threshold for selecting the number of clusters (0.15) is indicated by the red dashed line.

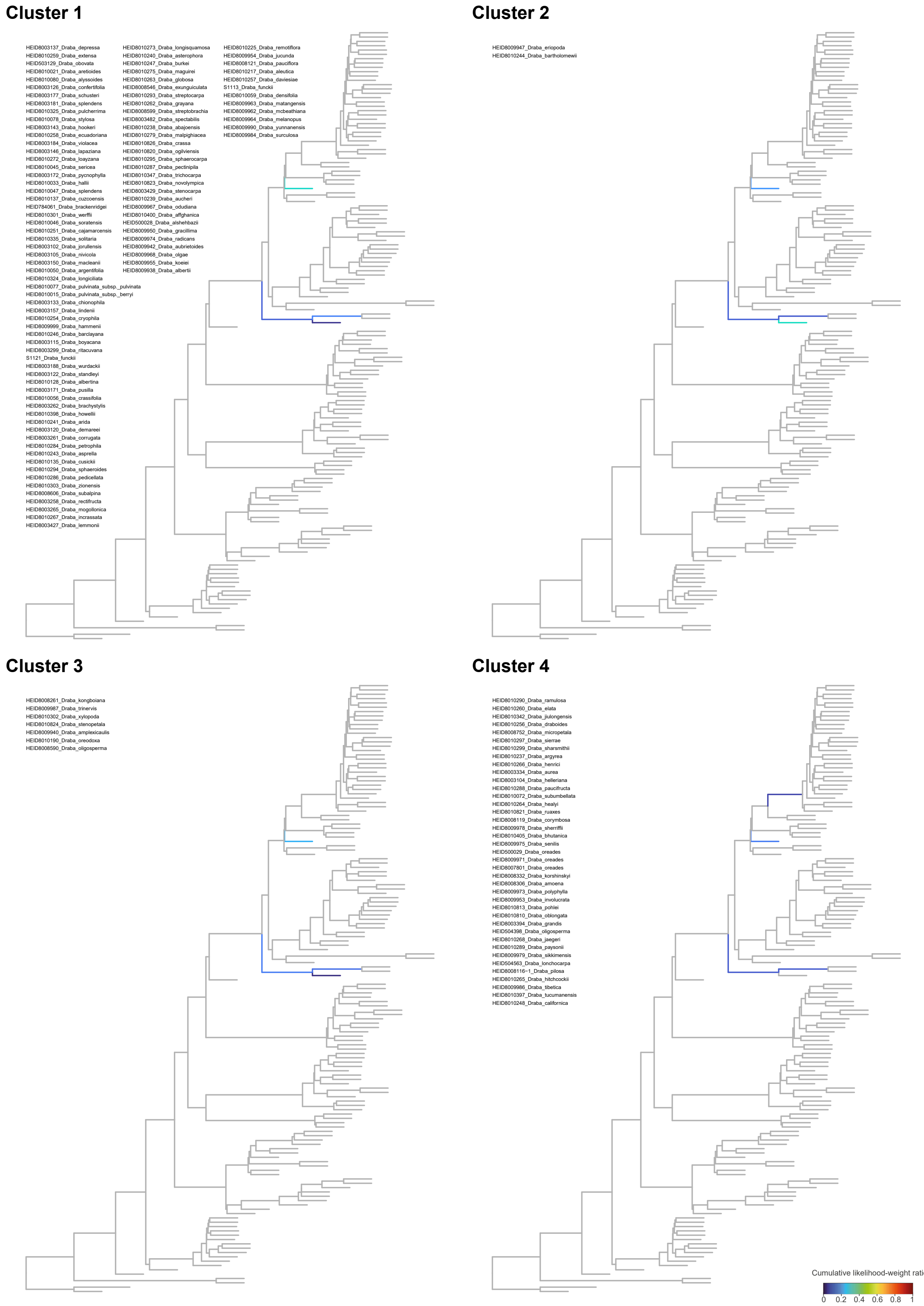

Figure S12. Dendrograms for gene copy placement of clusters 1-4. Diploid trees with branches colored according to placement proportion are shown; samples assigned to each cluster are given in the top left corner of each subplot. A threshold of 0.05 was chosen to display placement proportion.

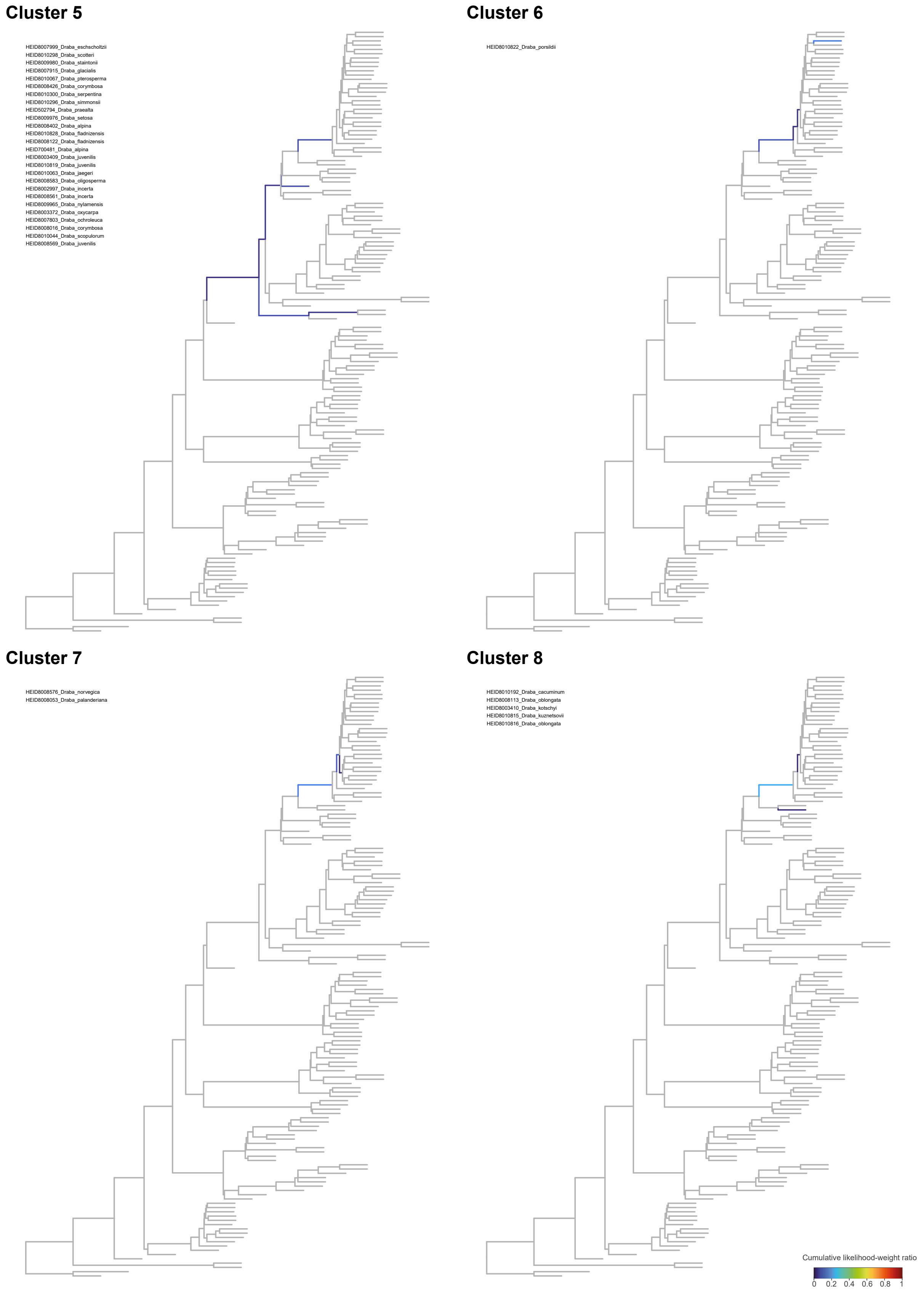

Figure S13. Dendrograms for gene copy placement of clusters 5-8. Diploid trees with branches colored according to placement proportion are shown; samples assigned to each cluster are given in the top left corner of each subplot. A threshold of 0.05 was chosen to display placement proportion.

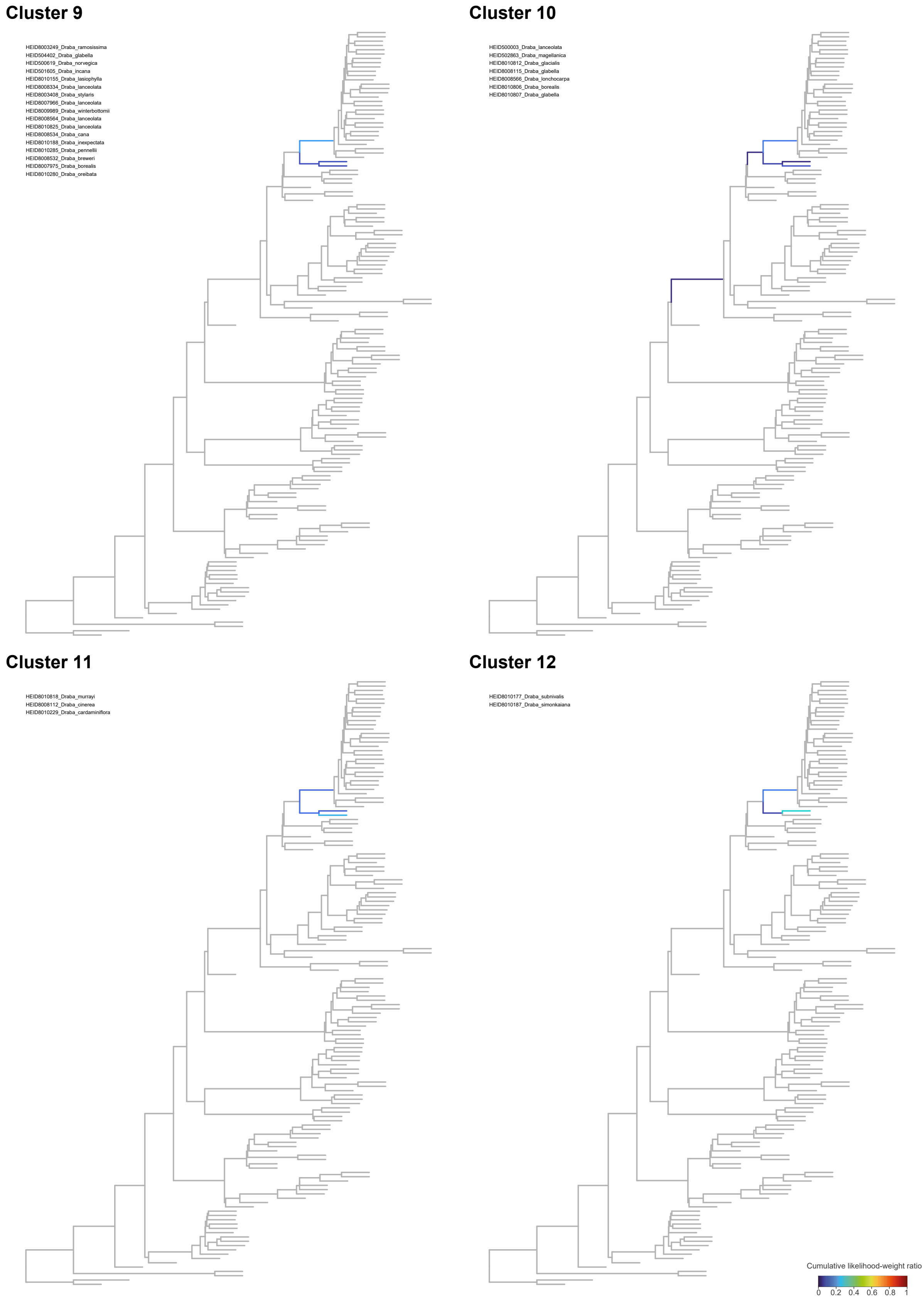

Figure S14. Dendrograms for gene copy placement of clusters 9-12. Diploid trees with branches colored according to placement proportion are shown; samples assigned to each cluster are given in the top left corner of each subplot. A threshold of 0.05 was chosen to display placement proportion.

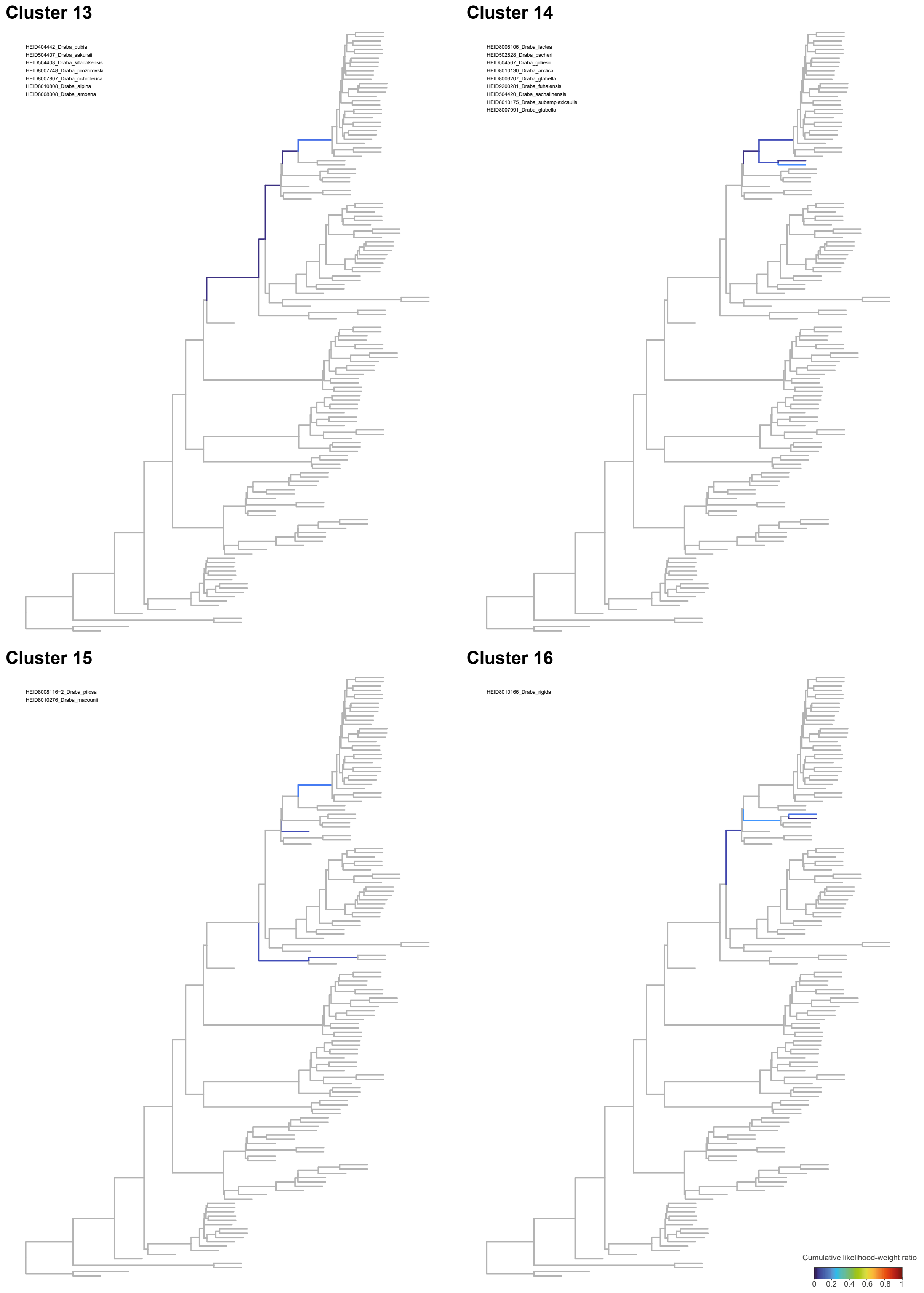

Figure S15. Dendrograms for gene copy placement of clusters 13-16. Diploid trees with branches colored according to placement proportion are shown; samples assigned to each cluster are given in the top left corner of each subplot. A threshold of 0.05 was chosen to display placement proportion.

Cluster 17

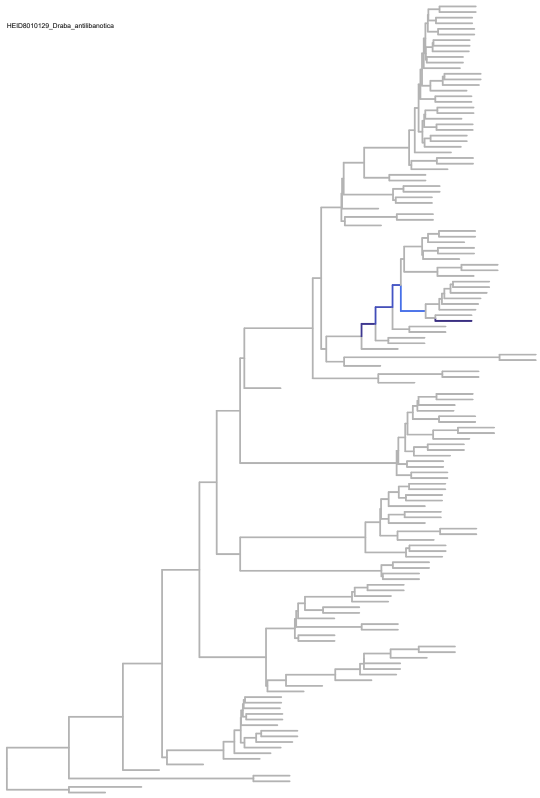

Cluster 18

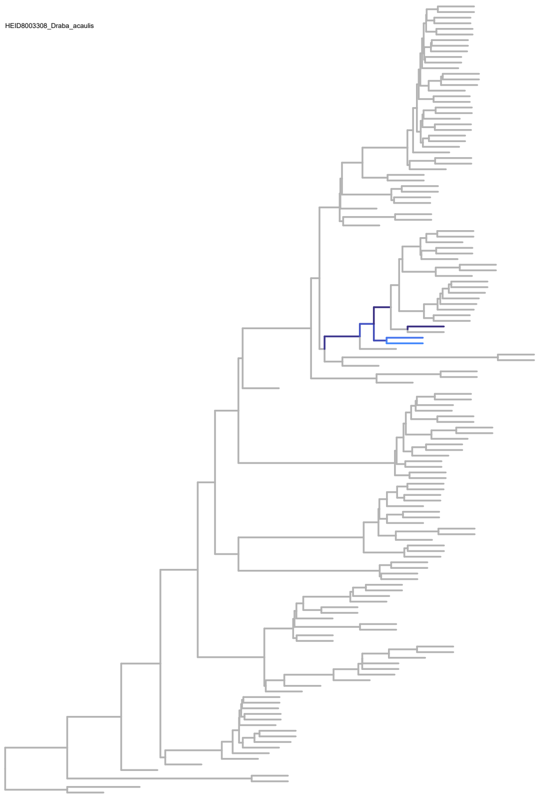

Cluster 19

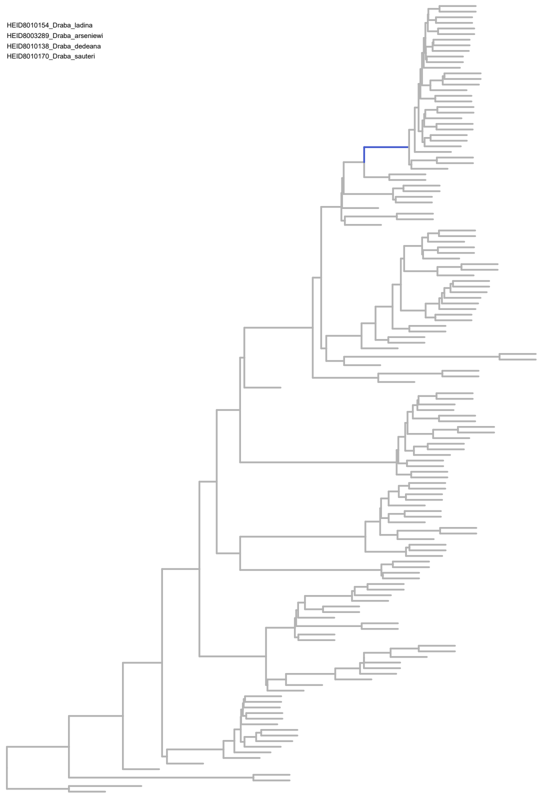

Cluster 20

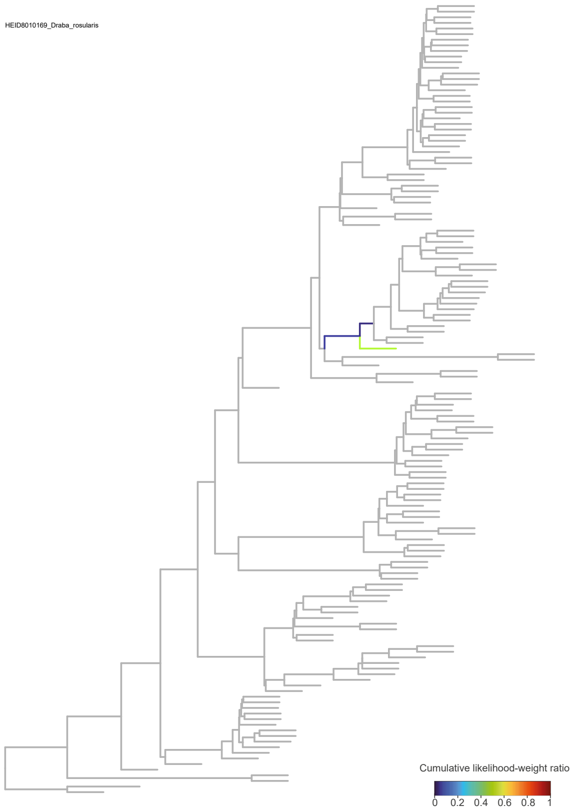

Figure S16. Dendrograms for gene copy placement of clusters 17-20. Diploid trees with branches colored according to placement proportion are shown; samples assigned to each cluster are given in the top left corner of each subplot. A threshold of 0.05 was chosen to display placement proportion.

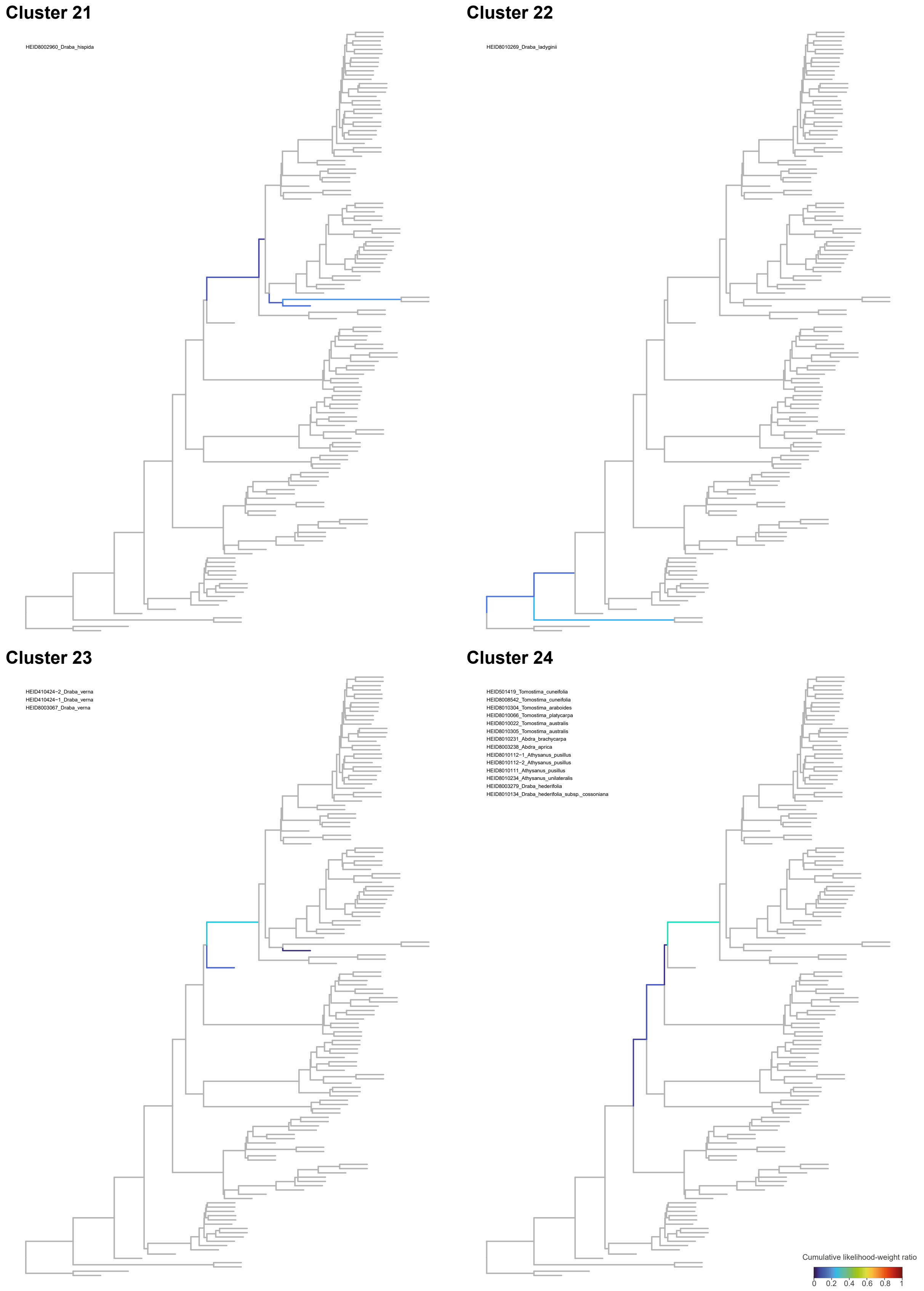

Figure S17. Dendrograms for gene copy placement of clusters 21-24. Diploid trees with branches colored according to placement proportion are shown; samples assigned to each cluster are given in the top left corner of each subplot. A threshold of 0.05 was chosen to display placement proportion.

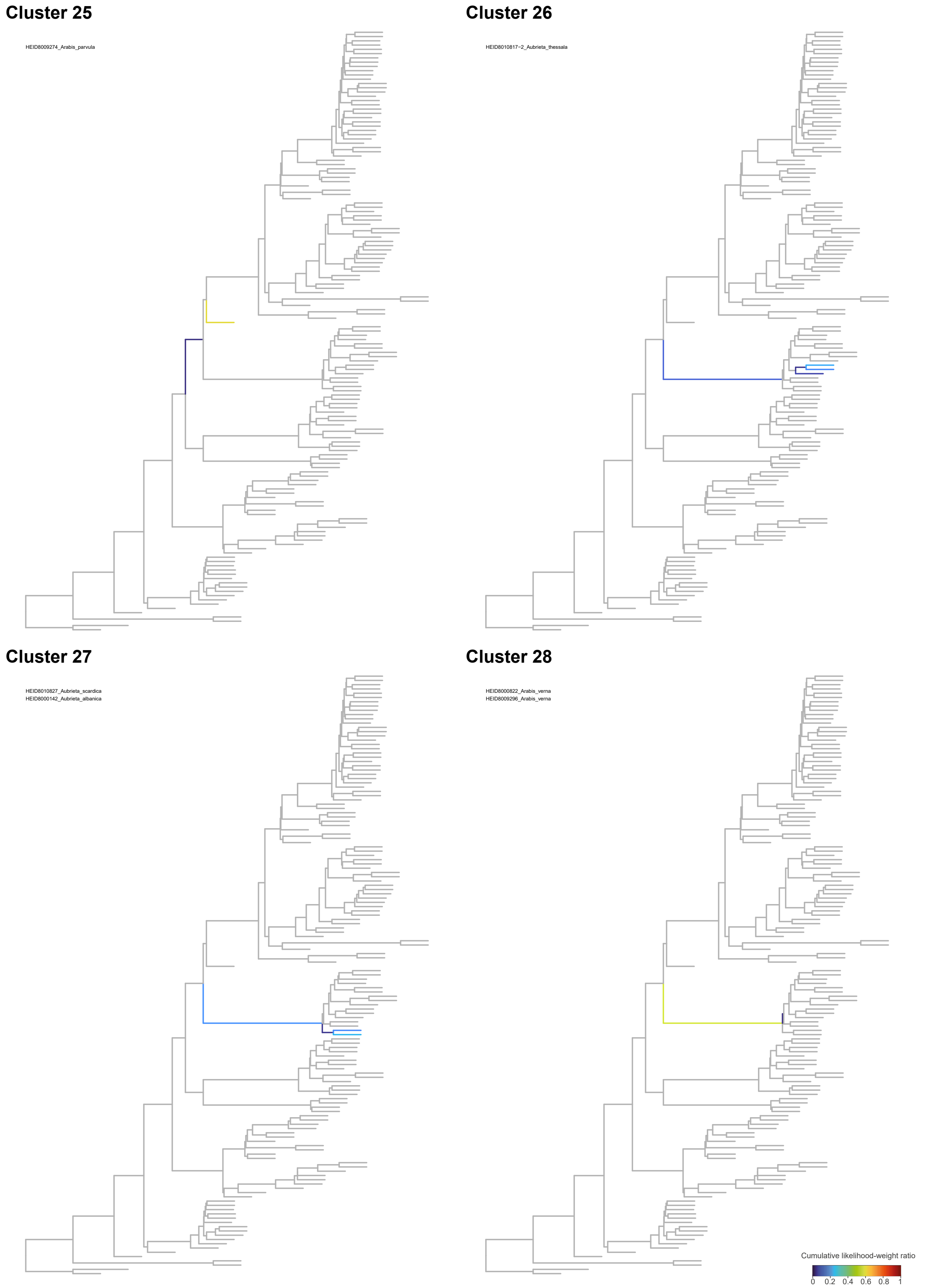

Figure S18. Dendrograms for gene copy placement of clusters 25-28. Diploid trees with branches colored according to placement proportion are shown; samples assigned to each cluster are given in the top left corner of each subplot. A threshold of 0.05 was chosen to display placement proportion.

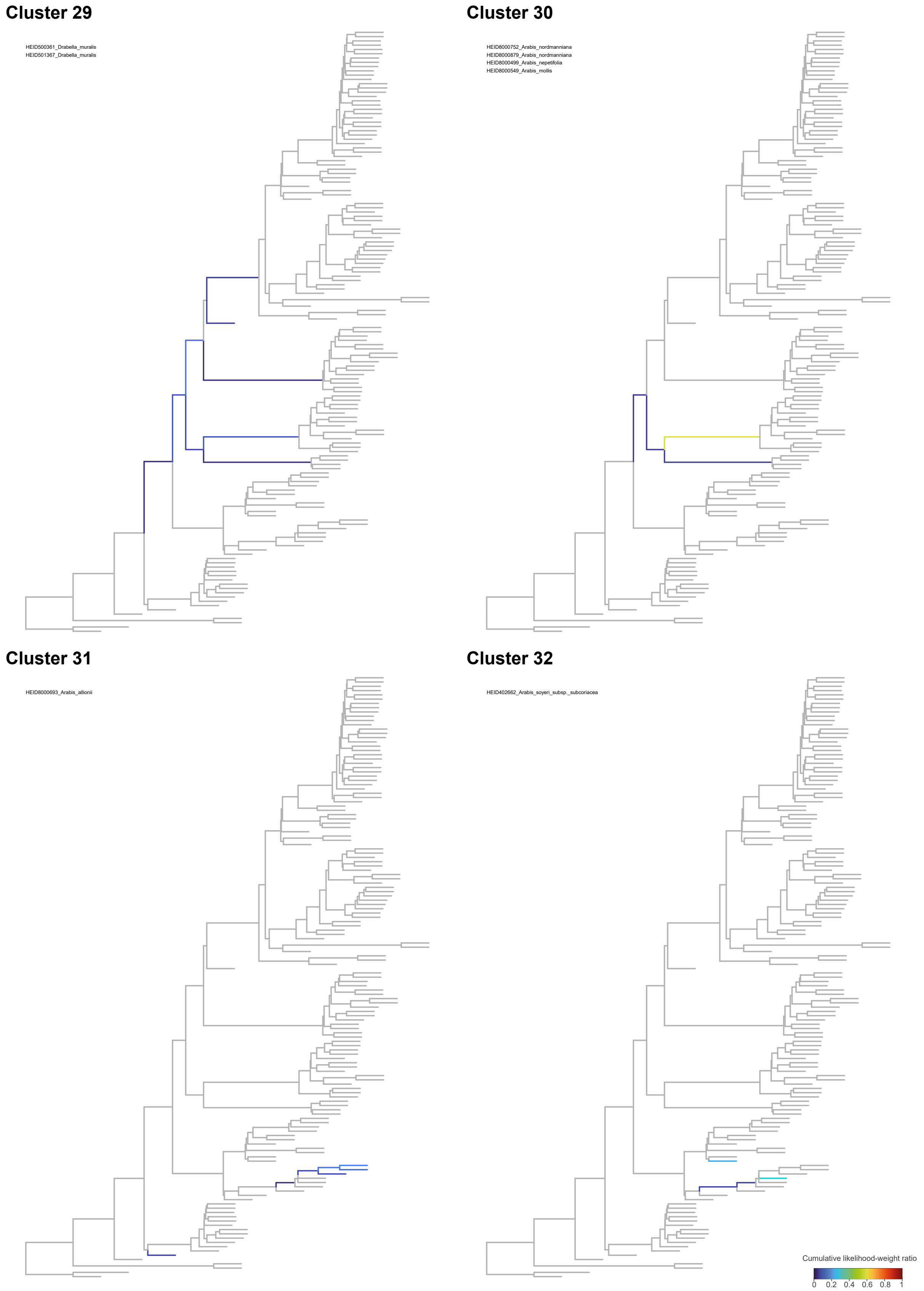

Figure S19. Dendrograms for gene copy placement of clusters 29-32. Diploid trees with branches colored according to placement proportion are shown; samples assigned to each cluster are given in the top left corner of each subplot. A threshold of 0.05 was chosen to display placement proportion.

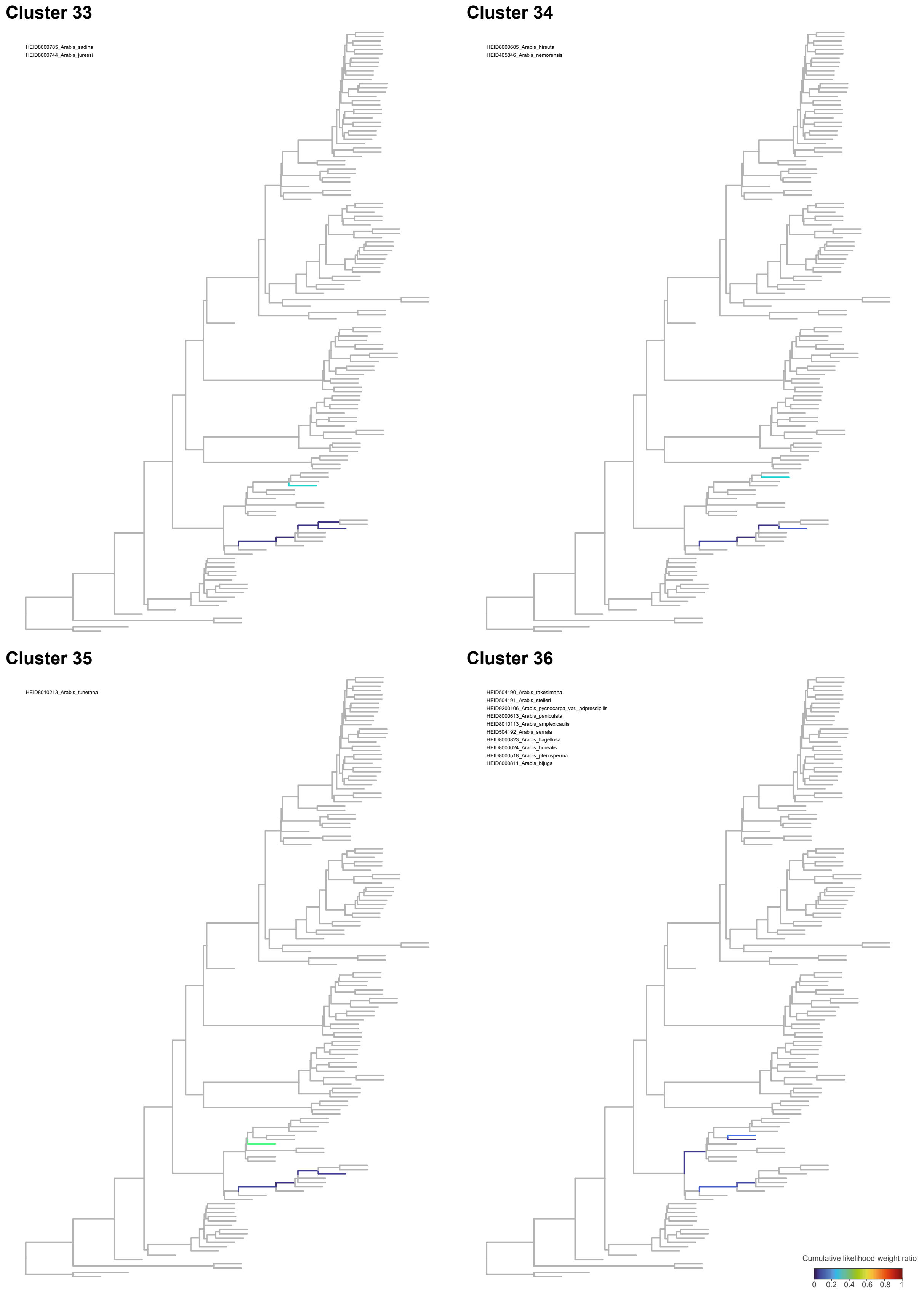

Figure S20. Dendrograms for gene copy placement of clusters 33-36. Diploid trees with branches colored according to placement proportion are shown; samples assigned to each cluster are given in the top left corner of each subplot. A threshold of 0.05 was chosen to display placement proportion.

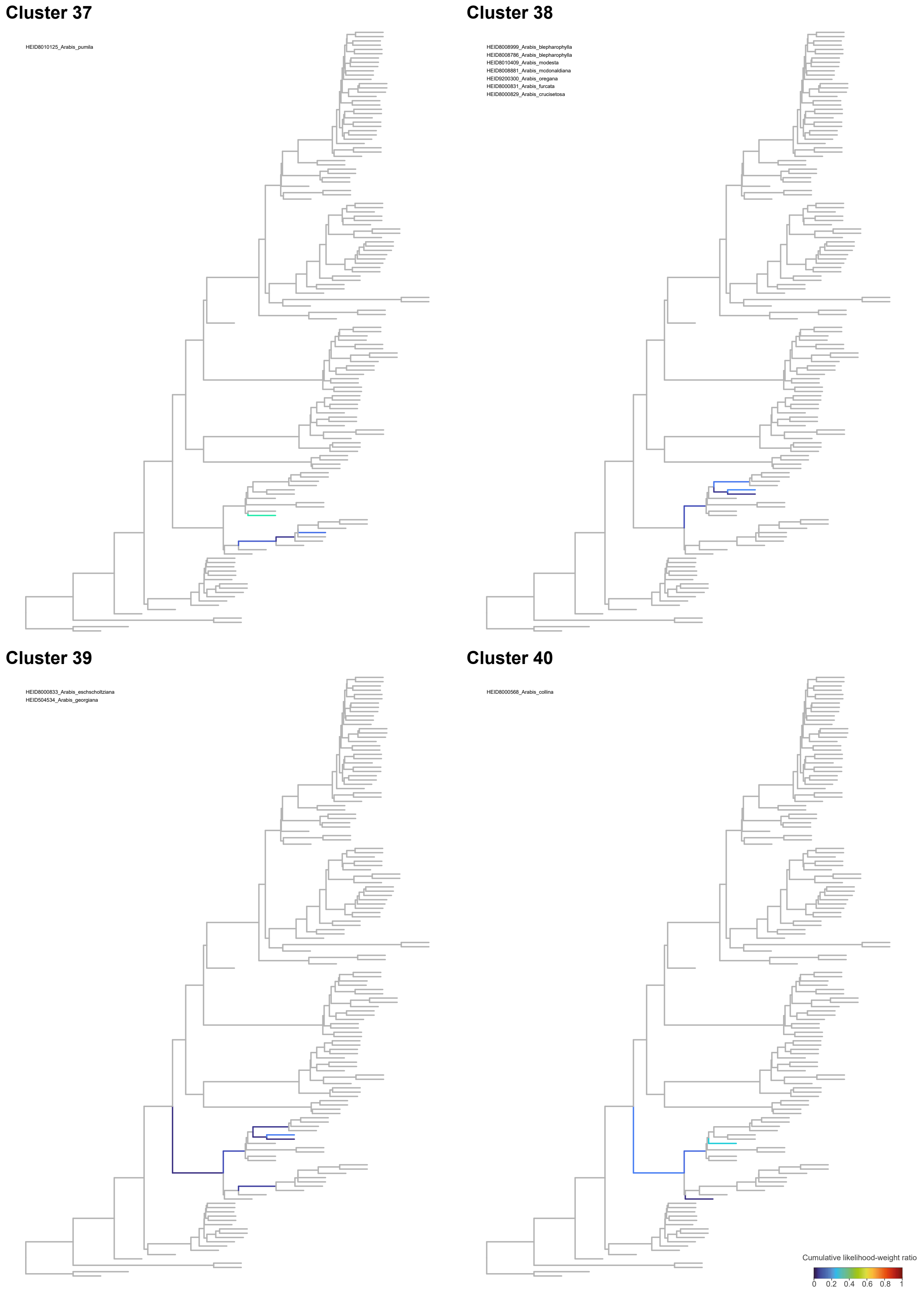

Figure S21. Dendrograms for gene copy placement of clusters 37-40. Diploid trees with branches colored according to placement proportion are shown; samples assigned to each cluster are given in the top left corner of each subplot. A threshold of 0.05 was chosen to display placement proportion.

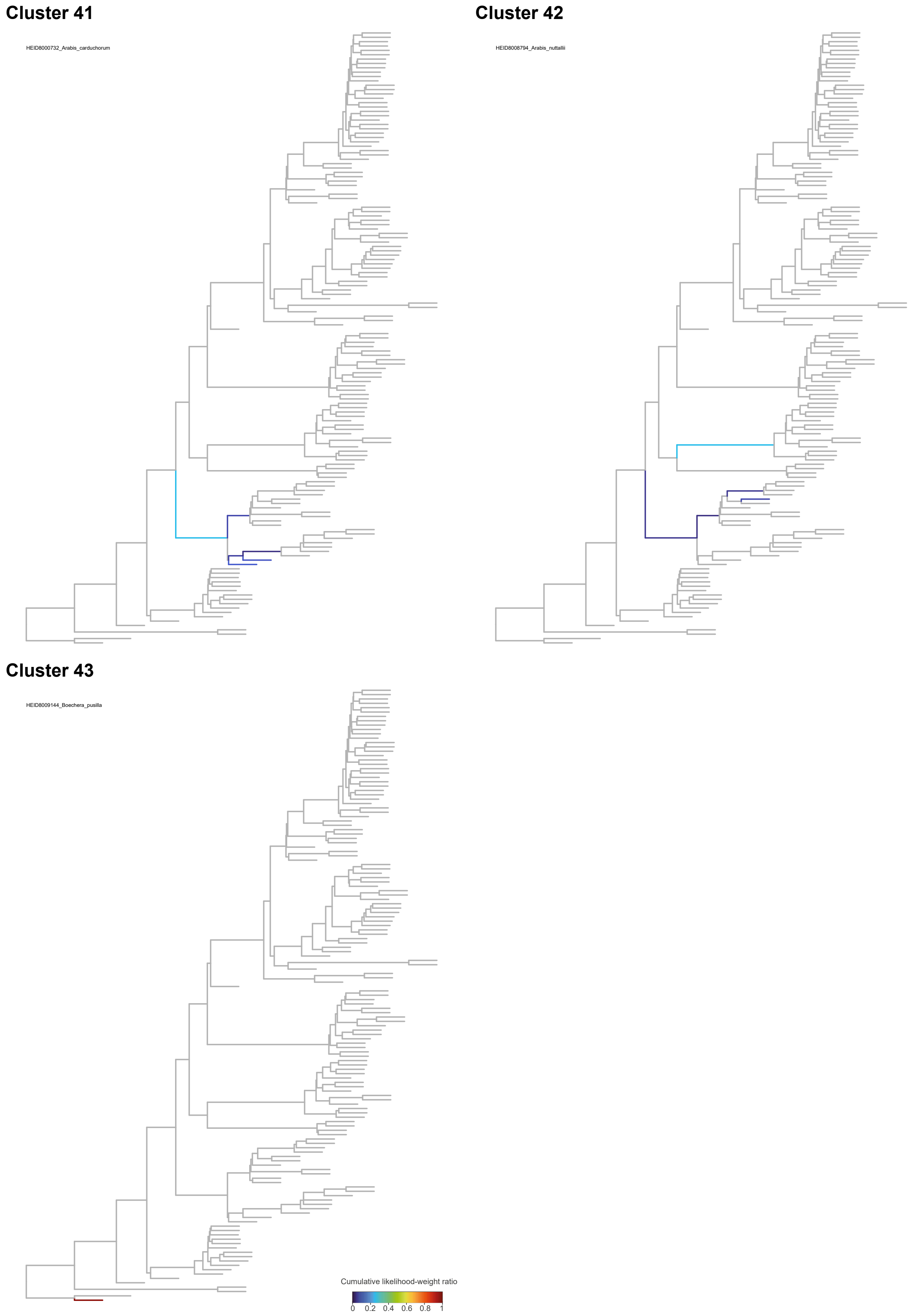

Figure S22. Dendrograms for gene copy placement of clusters 41-43. Diploid trees with branches colored according to placement proportion are shown; samples assigned to each cluster are given in the top left corner of each subplot. A threshold of 0.05 was chosen to display placement proportion.

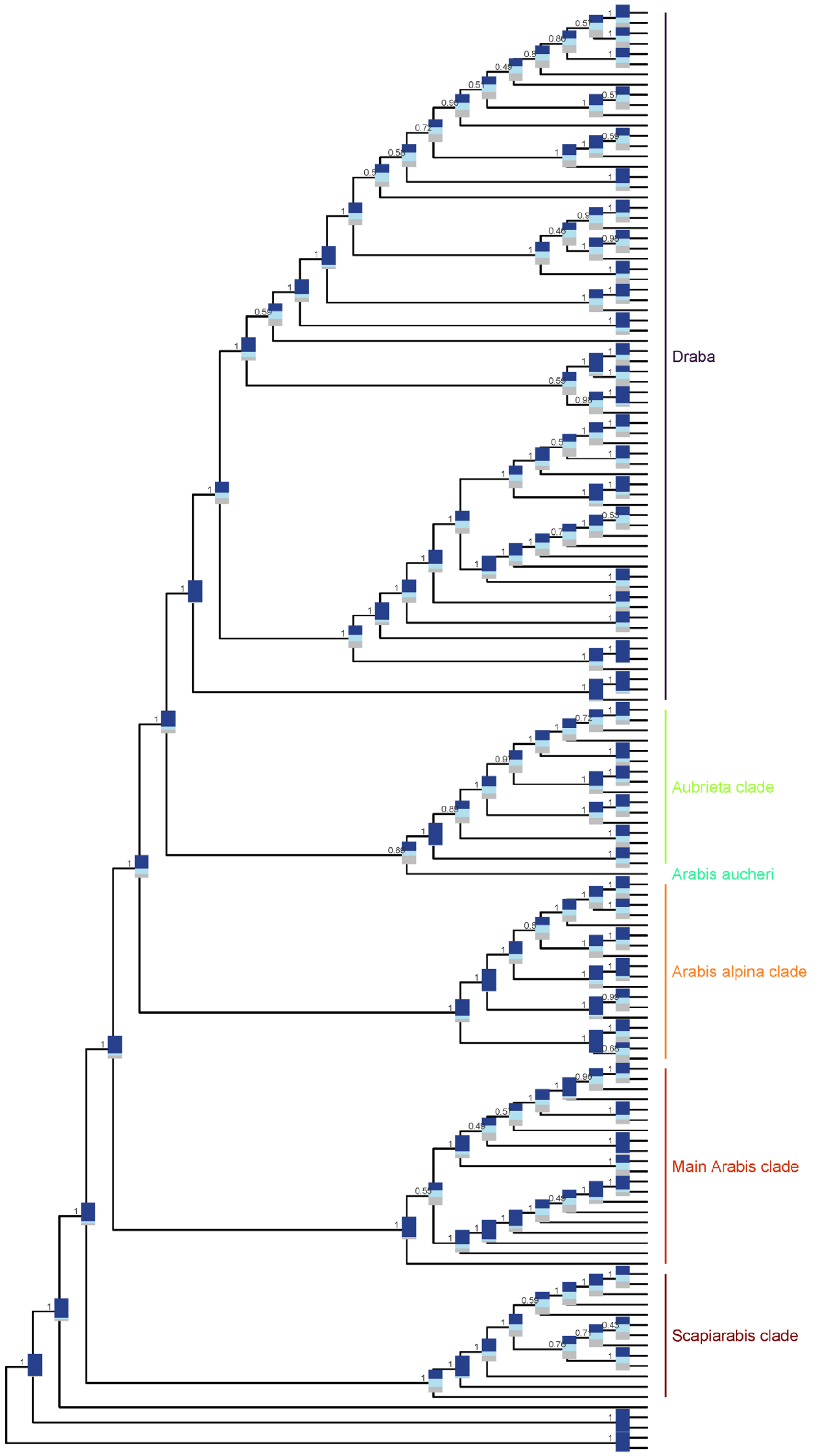

Figure S23. ASTRAL-pro tree based on individual exon copies from diploid samples. The tree was reconstructed from 567 gene trees that included all homologs assembled by HybPiper using a single exon per gene. Posterior probabilities for the first topology are shown as node labels and quartet scores are shown as bar charts: dark blue for the first quartet, light blue for the first alternative.

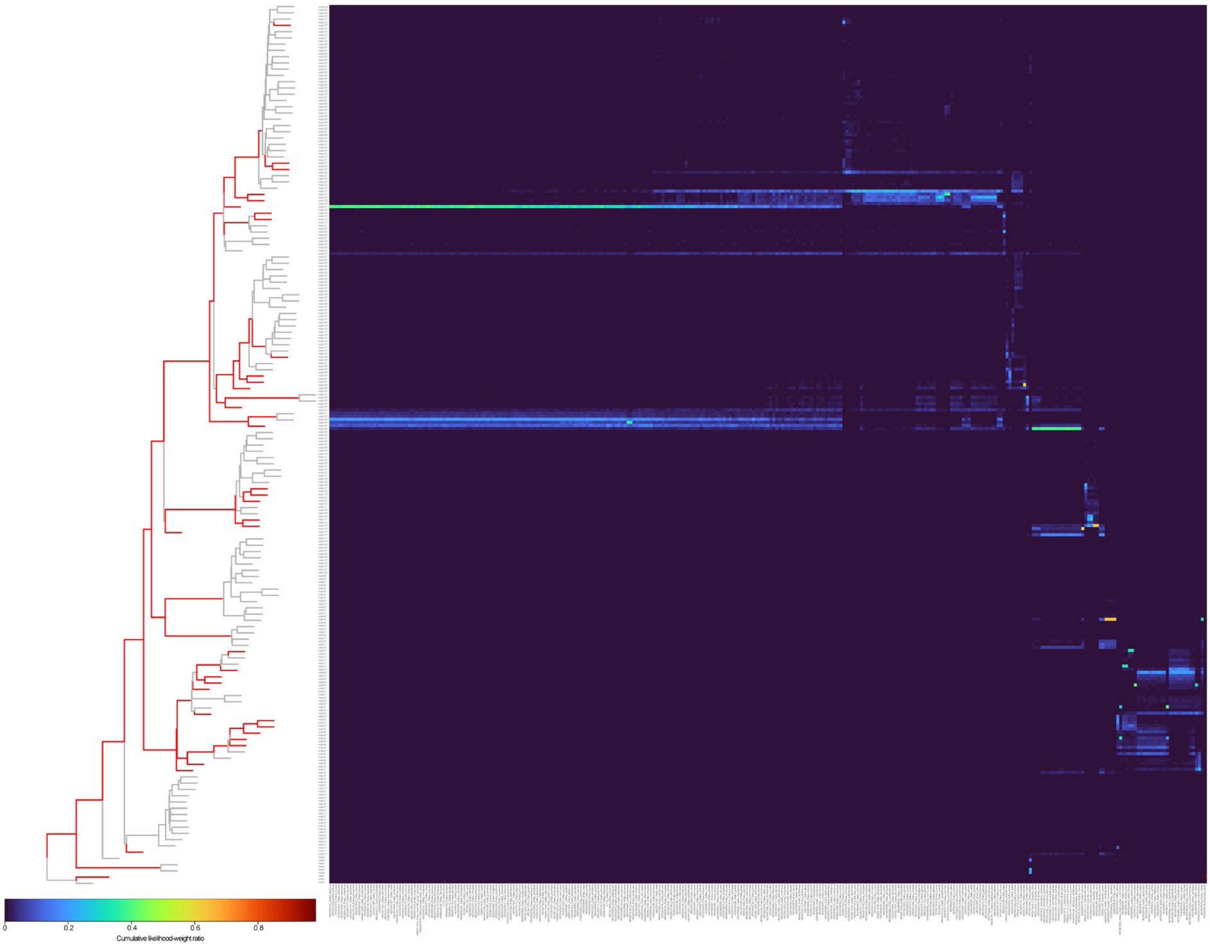

Figure S24. Phylogenetic placement for exon sequences. Cumulative likelihood-weight ratios (lwr) over all gene copies from ‘polyploid’ samples are given. Samples are arranged in the same order as in Figure 3 and Figure S11 on the x-axis, and edges in the tree are displayed on the y-axis. Note that the diploid anchor tree had a slightly different topology compared to the analysis with all 994 genes. The dendrogram of the diploid tree was added for orientation; edges with mean lwr ≥ 0.05 in at least one cluster are highlighted in red.

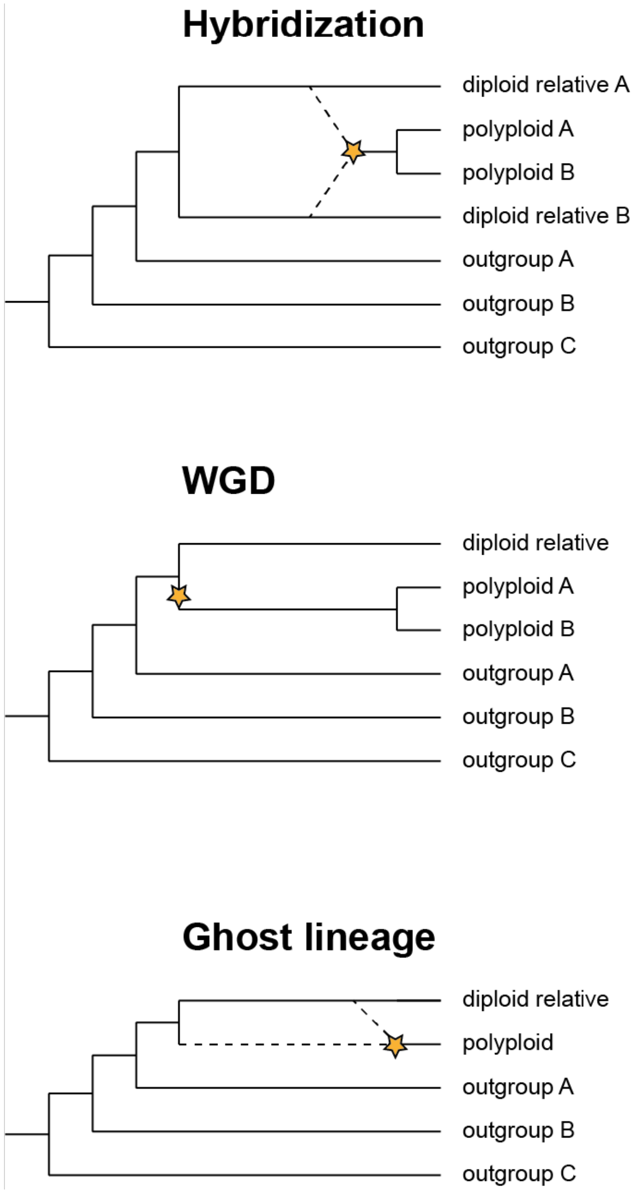

Figure S25. Three evolutionary scenarios for the emergence of polyploids analyzed with *Ks*. Hybridization (allopolyploidization) between two diploid lineages leading to the emergence of a polyploid lineage with subsequent lineage diversification, ancient whole-genome duplication (autopolyploidization) at the time of lineage divergence with subsequent lineage diversification, and emergence of a polyploid through recent hybridization of a diploid relative and a now extinct ghost lineage. The orange star symbolizes the emergence of the polyploid taxon/clade.

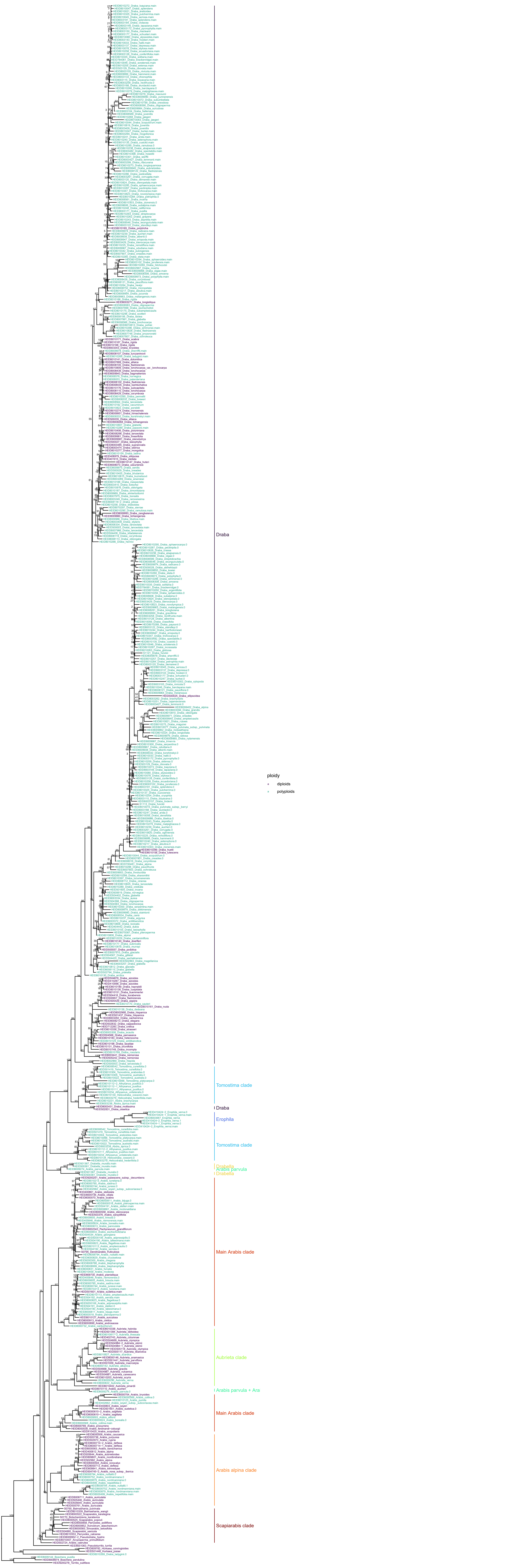

Figure S26. Multi-labeled gene tree. The phylogenetic tree was reconstructed from all gene copies of gene AT5G24240 using IQtree. Bootstrap support (based on 1000 UFboot replicates) is given as node labels, major evolutionary lineages are highlighted on the right. Ploidy is color coded using tip labels (diploids in purple, polyploids in green).
