# Supplementary material for: Unravelling Complex Hybrid and Polyploid Evolutionary Relationships Using Phylogenetic Placement of Homologous Gene Copies from Target Enrichment Data
Source: Syst Biol. 2026 Jan 27;75(6):1120–39. doi: 10.1093/sysbio/syag007 (PMC13637605; doi:10.1093/sysbio/syag007)
Supplement: Supplementary file 1 [file syag007_supplemental_files.zip › List_Supplementary_Tables_and_Figures.docx]

**Supplementary Tables and Figures**

Supplementary Text. New taxonomic combination *Turritis subflava*.

Table S1. Sample and sequencing information for Arabideae.

Table S2. Sample and sequencing information for *Arabidopsis*.

Figure S1. Mapping rate.

Figure S2. Gene recovery rate.

Figure S3. Gene selection.

Figure S4. Gene selection for phylogenetic placement.

Figure S5. Number of paralog warnings.

Figure S6. Detection of multiple gene copies by ploidy.

Figure S7. ASTRAL-pro tree based on gene copies from diploid samples.

Figure S8. Support values for ASTRAL-pro tree based on gene copies from diploid samples.

Figure S9. Phylogenetic placement.

Figure S10. Scatterplot of clustering height and number of clusters.

Figure S11. Dendrogram of hierarchical clustering.

Figure S12. Dendrograms for gene copy placement of clusters 1-4.

Figure S13. Dendrograms for gene copy placement of clusters 5-8.

Figure S14. Dendrograms for gene copy placement of clusters 9-12.

Figure S15. Dendrograms for gene copy placement of clusters 13-16.

Figure S16. Dendrograms for gene copy placement of clusters 17-20.

Figure S17. Dendrograms for gene copy placement of clusters 21-24.

Figure S18. Dendrograms for gene copy placement of clusters 25-28.

Figure S19. Dendrograms for gene copy placement of clusters 29-32.

Figure S20. Dendrograms for gene copy placement of clusters 33-36.

Figure S21. Dendrograms for gene copy placement of clusters 37-40.

Figure S22. Dendrograms for gene copy placement of clusters 41-43.

Figure S23. ASTRAL-pro tree based on individual exon copies from diploid samples.

Figure S24. Phylogenetic placement for exon sequences.

Figure S25. Three evolutionary scenarios for the emergence of polyploids analyzed with *Ks*.

Figure S26. Multi-labeled gene tree.
